# Ethyl ferulate contributes to the inhibition of the inflammatory responses in murine RAW 264.7 macrophage cells and acute lung injury in mice

**DOI:** 10.1371/journal.pone.0251578

**Published:** 2021-05-26

**Authors:** Yu Wang, Xuan Zhang, Linger Li, Zhao Zhang, Chengxi Wei, Guohua Gong

**Affiliations:** 1 Inner Mongolia University for Nationalities, Tongliao, Inner Mongolia, P.R. China; 2 Inner Mongolia Key Laboratory of Mongolian Medicine Pharmacology for Cardio-Cerebral Vascular System, Tongliao, Inner Mongolia, P.R. China; 3 Affiliated Hospital of Inner Mongolia University for Nationalities, Tongliao, Inner Mongolia, P.R. China; Xiangtan University, CHINA

## Abstract

**Background:**

Ethyl ferulate (EF) is a derivative of ferulic acid (FA), which is a monomeric component purified from the traditional medicinal herb *Ferula*, but its effects have not been clear yet. The purpose of this study was to evaluate whether EF can reduce inflammation levels in macrophages by regulating the Nrf2-HO-1 and NF-кB pathway.

**Methods:**

The LPS-induced raw 264.7 macrophage cells model was used to determine the anti-inflammatory and anti-oxidative stress effects of EF. The levels of IL-1β, IL-6, TNF-α and PGE2 were analyzed by ELISA. The mRNA and protein of COX-2, iNOS, TNF-α, IL-6, HO-1 and Nrf2 were identified by RT-PCR analysis and western blotting. Intracellular ROS levels were assessed with DCFH oxidation staining. The expressions of NF-кB p-p65 and Nrf2 were analyzed by immunofluorescence assay. The inhibitory effect of Nrf2 inhibitor ML385 (2μM) on mediatation of antioxidant activity by raw 264.7 macrophage cells was evaluated. The effect of EF was confirmed in acute lung injury mice model.

**Results:**

In our research, EF reduced the expression of iNOS, COX2 and the production of PGE2. EF could inhibit the production of pro-inflammatory cytokines (IL-1β, IL-6 and TNF-α) in lipopolysaccharide (LPS) stimulated macrophages and decreased expression of IL-6 and TNF-α in LPS stimulated macrophages. Furthermore, EF inhibited NF-кB p65 from transporting to the nucleus, decreased the expression of p-IкBα, significantly decreased the level of intracellular reactive oxygen species (ROS) and activated Nrf2/HO-1 pathways. EF could attenuate the degree of leukocyte infiltration, reduced MPO activity, mRNA levels and secretion of TNF-α and IL-6 in vivo. EF exhibited potent protective effects against LPS-induced acute lung injury in mice.

**Conclusions:**

Collectively, our data showed that EF relieved LPS-induced inflammatory responses by inhibiting NF-κB pathway and activating Nrf2/HO-1 pathway, known to be involved in the regulation of inflammatory responses by Nrf2.

## Introduction

Inflammation is triggered by harmful stimuli. A properly regulated inflammation can protect the host against infection, but persistent and recurrent episodes of inflammation mediated by aberrant activation of immunity lead to many inflammatory diseases, such as atherosclerosis, acute lung injury, inflammatory bowel disease and so on [1]. Therefore, a proper regulation of inflammation is believed to post a great challenging for human health. Macrophages is the main pro-inflammatroy cells and regulation of macrophage polarization may be a potential rational therapeutic target for acute lung injury. Macrophages release various proinflammatory meditors, such as tumor necrosis factor α (TNF-α) and interleukin 6 (IL-6) following lipopolysaccharide (LPS) stimulation.

The occurrence of inflammation is finely regulated by the expression of inflammation-related genes at multiple levels including transcription and phosphorylation. Signaling pathway of nuclear factor-kB (NF-kB), a transcription factor, is involved in the inflammatory responses [2]. Upon activation, NF-kB signaling pathway activates the expression of a limited set of transcription factors that promote the transcription of inflammatory genes, including pro-inflammatory cytokines such as tumor necrosis factor α (TNF-α) and interleukin 6 (IL-6), pro-inflammatory mediators like prostaglandins (PGs) secreted from the activated macrophages, which are a class of bioactive lipid mediators of an array of (patho) physiological conditions such as inflammation [3]. Cyclooxygenase (COX), a membrane-bound protein, is the rate limiting enzyme for PG biosynthesis. COX-2 can be induced rapidly in inflammatory cells by internal and external stimuli such as LPS [4].

During inflammatory responses, activated neutrophils and macrophage generate prodigious amount of reactive oxygen species (ROS) [5], which will bring about reversible or irreversible chemical changes in proteins, lipids and DNA, resulting in diminished biochemical functions [6]. In general, the excessive amount of ROS can damage cells and organs, cause a nuclear deficiency of NF-E2-related factor 2 (Nrf2) by impairing its ability of translocating to nucleus as reduced ubiquination.

Ferulic acid (4-hydroxy-3-methoxycinnamic acid, FA) belongs to the phenolic acid group commonly found in *Ferula*, *Angelica sinensis* and so on. It has been shown that FA has a wide range of pharmacological properties including anti-oxidative, anti-inflammatory and neuro-protective [7]. Due to charge delocalization between the aromatic ring and the double bond in the side chain, the efficiency of FA has been linked to the stability of its phenoxyl radical [8]. Some derivatives of FA, such as Ethyl ferulate (EF), is demonstrated with stronger activity and lower toxicity [9]. EF presents in various systems of many plants, such as the solanaceas family as a trace contituent, which is more lipophilic and has higher cell membrane and blood-brain barrier permeability than FA. As its beneficial heath properties, EF has been widely studied. EF could protect brain cells by inhibiting oxidative as a potent inducer of HO-1 [10], and significantly inhibit the activity of NF-kappaB in lipopolysaccharide (LPS) stimulated RAW 264.7 macrophages [11]. These functions indicate that EF may have beneficial effects on inflammation by decreasing the activation of NF-kappaB. However, the specific anti-inflammatory mechanism of EF is not clear. In this study, the anti-inflammation effect of EF was analyzed in RAW 264.7 macrophages induced by LPS and acute lung injury in mice and the possible mechanism of anti-inflammation effect of EF was examined in RAW 264.7 macrophages.

## Materials and methods

### Cell culture and drug treatment

The raw 264.7 macrophage cells were obtained from Institute of Basic Medical Sciences Chinese Academy of Medical Sciences and maintained in high-glucose Dulbecco’s modified Eagle’s medium (DMEM) supplemented with 10% fetal bovine serum (FBS, PAN-Biotech, Aidenbach, Germany) and 1% penicillin-streptomycin solution at 37°C in a humidified atmosphere of 95% air and 5% CO_2_. All experiments were performed with cells on passages 6 to 10. LPS (1μg/mL, Sigma-Aldrich Chemical, Darmstadt, Germany, SMB00704) was applied for 24 h as mimicking inflammatory situation, and then different concentrations (10, 20, 40, 50, 80 mg/L) of EF were applied.

### Cell viability assay

Cytotoxicity of raw 264.7 macrophage cells were tested by the colorimetric assay with 3-(4, 5)-dimethylthiahiazo (-z-yl)-3, 5-di-phenytetrazoliumromide (MTT) (Sigma-Aldrich Chemical, Darmstadt, Germany, 11465007001). After incubation, cells were incubated by 10μL MTT (5 mg/mL) for 4 h. The medium was removed and 150 μL DMSO was added. The formazan dye crystals were solubilized for 10 min, and absorbance (wavelength: 570 nm) was measured by Thermo Scientific Microplate Reader.

### Animals

Male C57BL/6 mice were provided by YiSi Co. Ltd. (Changchun, China). They were watered ad libitum and housed in 12-h light/dark cycle at the temperature of 22 ± 2°C. All mice were fed a standard diet. Animal care and study protocols were approved by the Animal Care and Use Committee of Inner Mongolia University for the Nationalities. Mice were randomly assigned into 4 groups (*n* = 8/each group): control, LPS (0.5 mg/kg), LPS+EF (15mg/kg), LPS+EF (30mg/kg). Mice were intraperitoneally injected with two doses (15 and 30 mg/kg) of EF with vehicle twice a day for 5 day, then treated with LPS by nasal drip 1h later. Mice were killed 8 h after LPS treatment under anaesthesia by intraperitoneal injection of sodium pentobarbital with bronchoalveolar lavage fluid (BALF) and tissue samples collected.

### Enzyme-linked immunosorbent assay

The supernatants were collected and assayed to determine the levels of IL-1β, IL-6, TNF-α and PGE2 by an enzyme-linked immunosorbent assay (ELISA) in accordance with the manufacturer’s instruction (Biolegend, USA, (IL-1β) 432604 (IL-6) 431316, (TNF-α) 575209) and (solarbio, China (PGE2)SEKM-0173).

### RT-PCR analysis

Total RNAs were isolated from raw 264.7 macrophage cells with RNA extraction kit (TIANGEN, Beijing, China, DP430). CON, A260/280:1.99, A260/230:2.13, LPS, A260/280:2.01, A260/230:2.18, LPS+EF, A260/280:1.89, A260/230:2.23, A260/280:2.02, A260/230:2.37. And after their transcription, the obtained cDNA was used for real-time PCR. Primers were designed with Primer Premier 5.0 and were as follows:

COX-2-F, 5’-TGTGACTGTACCCGGACTGG-3’;

COX-2-R, 5’-TGCACATTGTAAGTAGGTGGAC-3’;

iNOS-F, 5’-GACAAGCTGCATGTGACATC-3’;

iNOS-R, 5’-GCTGGTA GGTTCCTGTTGTT-3’

TNF-α-F, 5’-CCCTCACACTCAGATCATCTTCT-3’;

TNF-α-R, 5’-GCTACGACGTGGGCTACAG-3’;

IL-6-F, 5’-ATGAAGTTCCTCTCTGCAAGAGACT-3’;

IL-6-R, 5’-CACTAGGTTTGCCGAGTAGATCTC-3’;

HO-1-F, 5’-CGCCTTCCTGCTCAACATT-3’;

HO-1-R, 5’-TGTGTTCCTCTGTCAGCATCAC-3’

GAPDH-F, 5’-CGACTTCAACAGCGACACTCAC-3’;

GAPDH-R, 5’-CCCTGTTGCTGTAGCCAAATTC-3’;

Amplification was performed in duplicate on StepOnePlus Real-Time PCR System Real-Time PCR system thermocycler by using SYBR Green PCR Master Mix (TIANGEN, Beijing, China, FP205). The reaction condition was 95°C for 15 min and following 40 cycles: denaturation (95°C for 10 s), annealing and elongation (60°C for 60 s). The results were normalized with GAPDH mRNA.

### Cytosolic and nuclear protein extraction

Cytosolic and nuclear extracts were prepared from raw 264.7 macrophage cells with the Nuclear and Cytoplasmic Protein Extraction Kit (Beyotime, Jiangsu, China, P0027) according to manufacturer’s instructions. In brief, cytosolic extracts were prepared by repeating cycles of freezing and thawing in 0.2 ml of cold buffer A. Nuclear protein was extracted with ice-cold buffer C.

### Western blot analysis

Western blot was performed as described previously [12]. Antibodies for anti-COX2 (1:1000, 12282), anti-iNOS (1:1000, 13120), anti-IL-6 (1:1000, 12912), anti-TNF-α (1:1000, 11948), anti-Nrf2 (1:1000, 12721), anti-p65 (1:1000, 8242), anti-HO-1 (1:1000, 86806), anti-p- IкBα (1:1000, 5209), and anti- IкBα (1:1000, 4814) were purchased from Cell Signaling Technology. Protein was extracted and mixed in loading buffer, and then equal amounts were fractionated on gel and transferred onto Hybond-C Extra nitrocellulose membrane with a semidry transfer apparatus. At last the protein was blocked with nonfat dry milk, adding antibodies, and was detected with supersignal west pico chemiluminescent substrate.

### Measurement of intracellular ROS

Intracellular ROS levels were assessed with DCFH oxidation staining (Beyotime, Jiangsu, China, S0033S). Briefly, raw 264.7 macrophage cells were seeded in 24 well plates and endured all sorts of treatments. Then, 5 μM fresh DCFH-DA (10mM stock solution) in PBS was utilized to incubate cells at 37°C for 30 min away from light. A fluorescence microscopy (LEICA DMi8, Weztlar, Germany) was used to visualize.

### Immunofluorescence assay

RAW264.7 cells were seeded in 24-well plates with a density of 1.5×10^5^ cells per well and cultured overnight. RAW264.7 cells were fixed using 4% p-formaldehyde for 20 min at room temperature, and the cell membranes were permeabilized by 0.5% Triton X-100 in cold PBS for 10 min and then blocked for 60 min with 5% BSA prepared in 0.1% Tween 20 (PBST). Afterward, Cells were then incubated at room temperature for 2 h in a5% BSA solution that contained specific primary antibodies (1:100). After washing, Alexa Fluor 488-labeled IgG secondary antibody was added at 1:1000 dilution in 5% BSA solution for 1 h, and then incubated under dark. After that, cells were stained with 25 μg/mL of 4-6-diamidino-2-phenylindole (DAPI) in PBST, examined by confocal fluorescence microscopy.

### Histopathological study

Mice were sacrificed in deep anesthesia, and the lungs of mice were excised and rinsed several times. Lung tissues were fixed with 4% paraformaldehyde, embedded in paraffin and cut. Hematoxylin and eosin (HE) staining was used to evaluate basic pathological analysis.

### Measurement of myeloperoxidase (MPO) in lung tissues

The collected lung tissues were homogenized and dissolved in extraction buffer to identify MPO activity by commercial kits to test the accumulation of neutrophils.

### Statistical analysis

Experimental results were shown with Means ± SEM, using SSPS17.0 statistical analysis software for data analysis, two groups using independent t test, multiple groups using one-way ANOVA test was used following ANOVA. The number of stars (*/#) indicates the p value range: *p value <0.05, **p value <0.01; #p<0.05, ##p<0.01.

## Results

### EF no cellular toxicity on raw 264.7 macrophage cells

**Fig 1:**
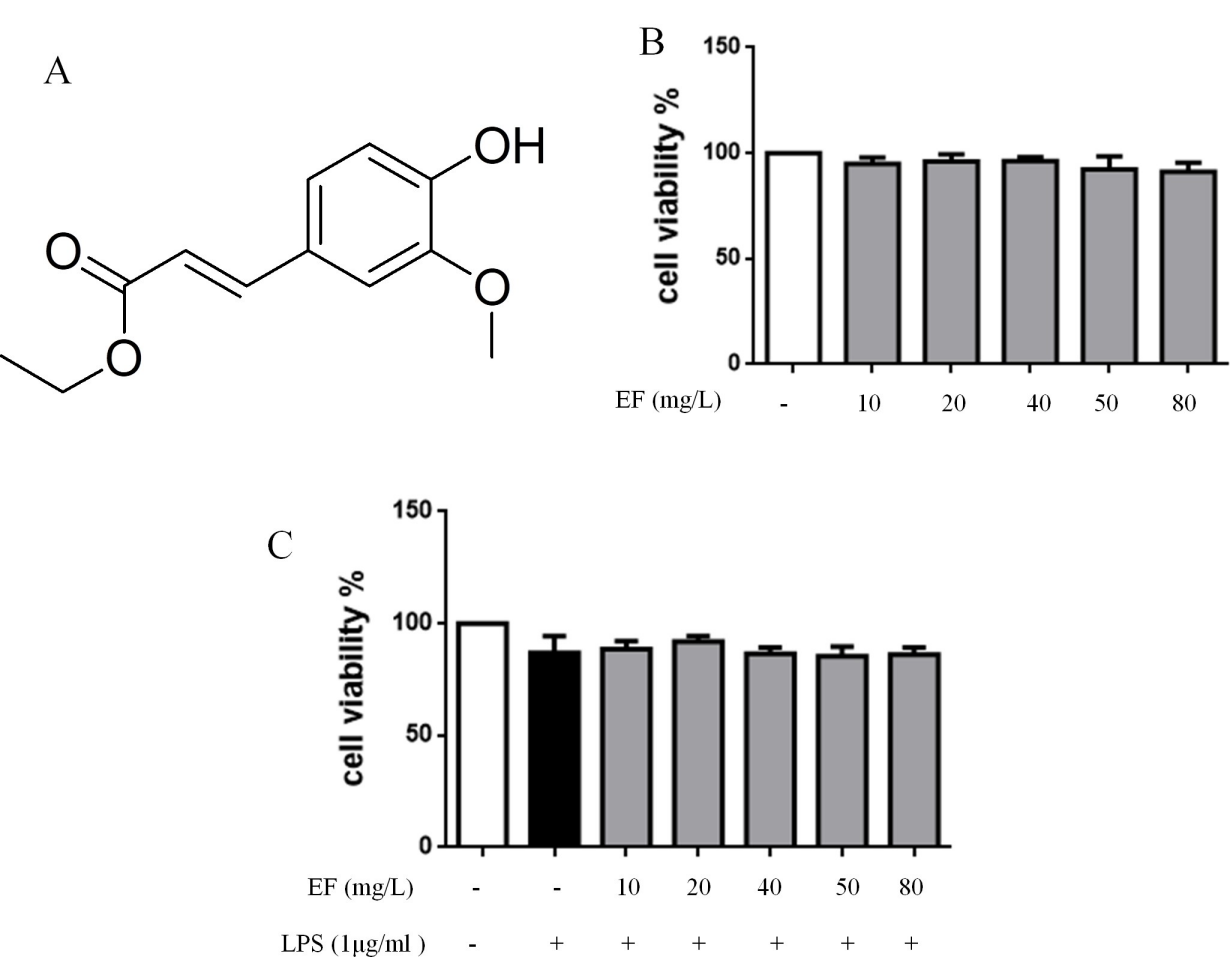
EF no cellular toxicity on raw 264.7 macrophage cells with or without LPS A: chemical structure of EF. B, C: cytotoxicity in cells treated with LPS with or without EF (10, 20, 40, 50, 80 mg/L).

The molecular chemical structure of EF was shown in Fig 1A. Raw 264.7 macrophage cells were cultured in LPS (1 μg/mL) with or without EF in different concentrations (10, 20, 40, 50, 80 mg/L), then their viability was tested by MTT assay to detect the cytotoxicity of EF. From Fig 1B and 1C, we found that EF at the concentration up to 80 mg/L had no cellular toxicity on raw 264.7 macrophage cells with or without LPS.

### EF inhibits production of PGE2 and expression of iNOS and COX2 induced by LPS

**Fig 2:**
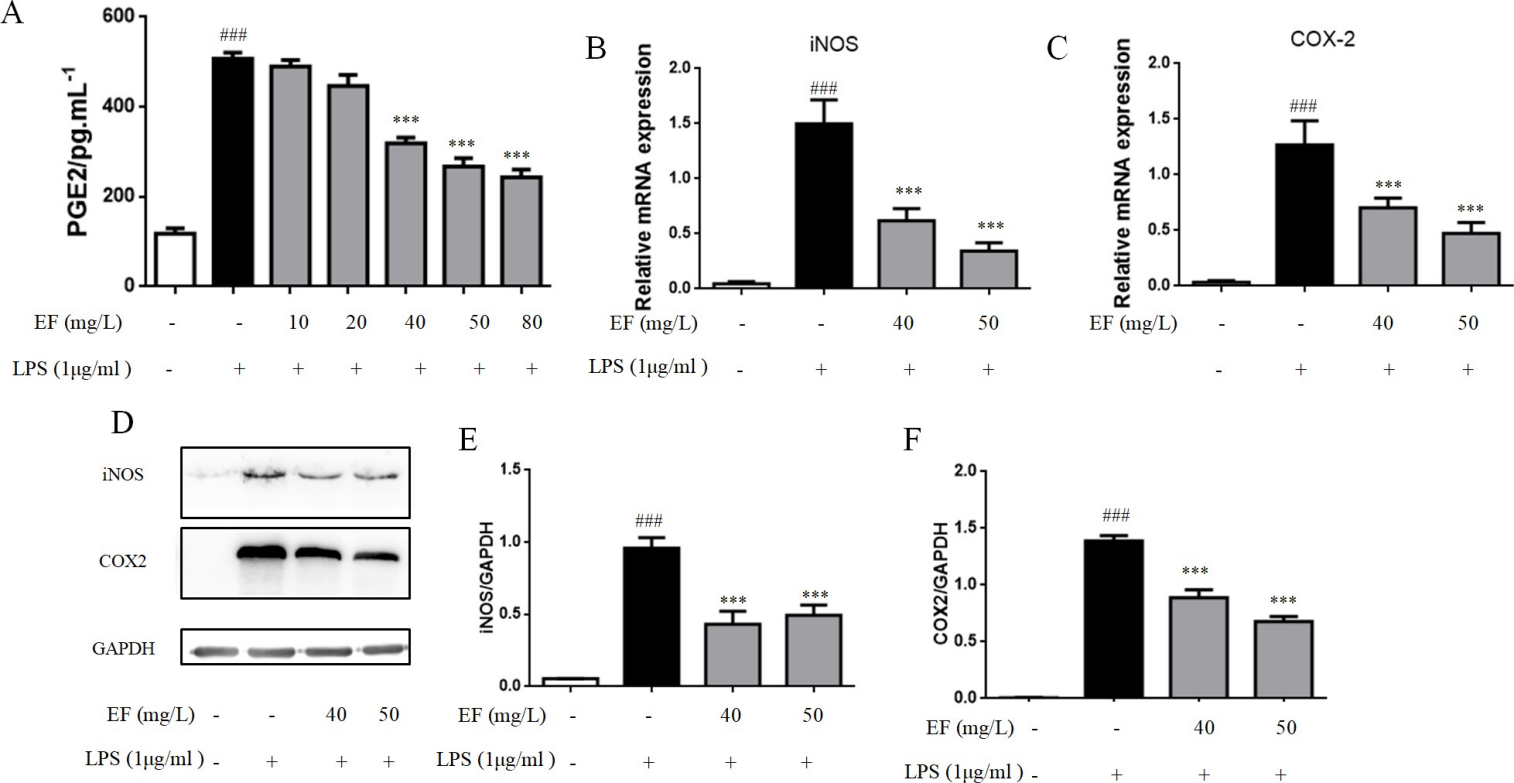
EF abrogated LPS stimulated production of PGE2 and inhibited the mRNA and protein levels of iNOS and COX-2. A: cells were treated with LPS (1 μg/ml) and then added EF (10, 20, 40, 50, 80 mg/L). PGE2 levels were analyzed by ELISA. B/C: The mRNA levels of iNOS and COX-2 were determined by quantitative real-time PCR. D/E/F: The protein level of iNOS and COX-2 was determined by Western blotting analysis using specific antibodies. All data are shown as mean ± SEM (n = 6, per group) ##P<0.01 vs control; **P<0.01 vs LPS.

To investigate the effect of EF on LPS-induced PGE2 production, cells were stimulated with LPS (1 μg/mL) for 24 h, then treated with EF in different concentrations (10, 20, 40, 50, 80 mg/L) for 24 h. As shown in Fig 2A, EF significantly inhibited the production of PGE2 in raw 264.7 macrophage cells stimulated by LPS (p value <0.01).

Raw 264.7 macrophage cells were treated with LPS with or without EF, and their iNOS and COX2 expression were analyzed by RT-PCR to analyze the effects of EF on them. The levels of iNOS and COX2 mRNA were markedly inhibited by treatment with EF (p value <0.01) (Fig 2B and 2C). In addition, western blotting analysis showed that treatment with EF inhibited LPS-stimulated expression of iNOS and COX2 expression (p value <0.01) (Fig 2D–2F).

### Inhibitory effect of EF on pro-inflammatory cytokine secretion in LPS-exposed raw 264.7 macrophage cells

**Fig 3:**
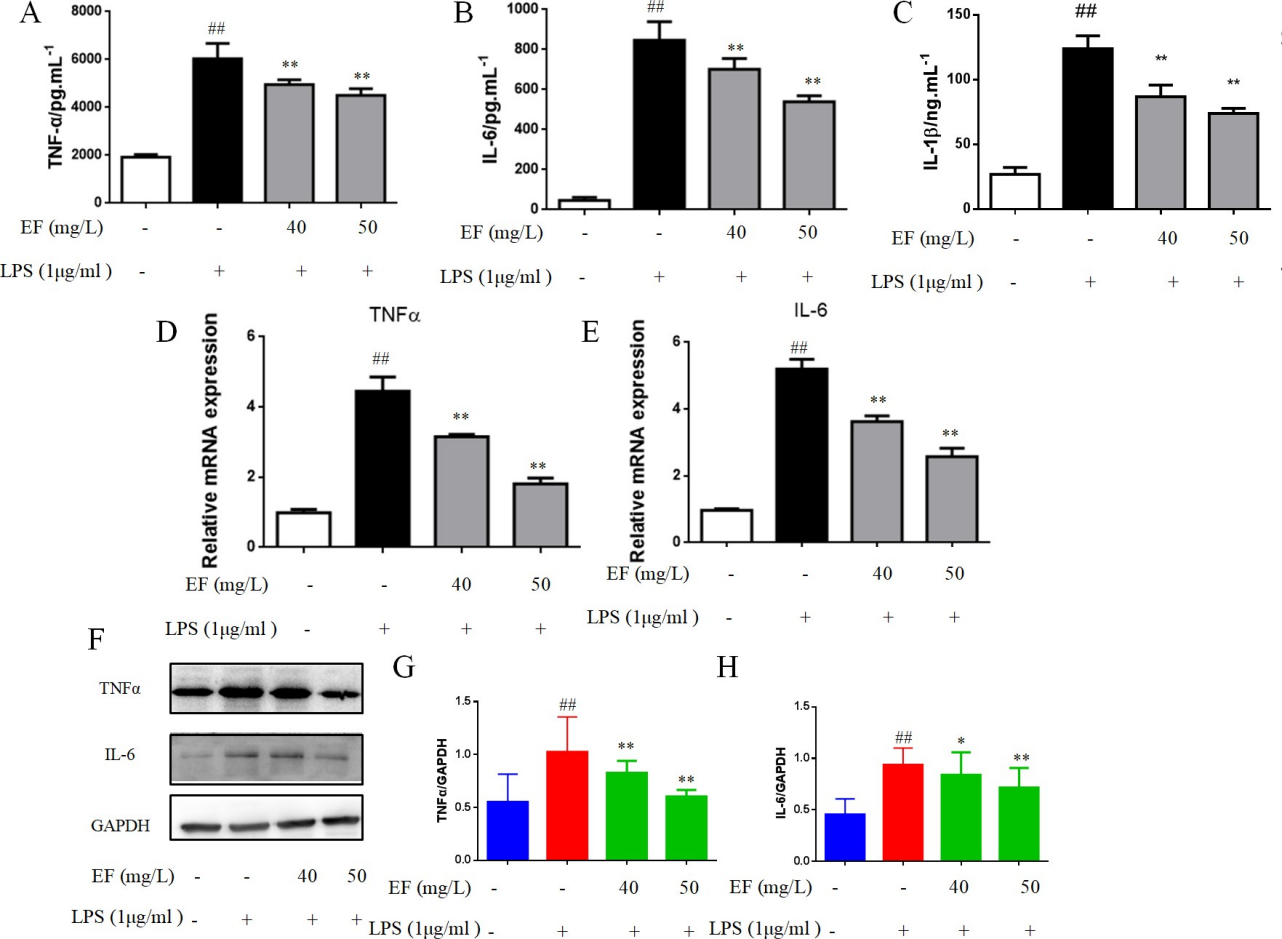
EF decreased LPS-stimulated expression of pro-inflammatory cytokines in RAW 264.7 macrophages cells. A/B/C: the supernatant levels of TNF-α, IL-6 and IL-1β were identified by ELISA. D/E: The mRNA levels of TNF-α and IL-6 were determined by quantitative real-time PCR. F/G/H: The protein level of TNF-α and IL-6 was determined by Western blotting analysis using specific antibodies. All data are shown as mean ± SEM (n = 6, per group) ##P<0.01 vs control; *P<0.05, **P<0.01 vs LPS.

The pro-inflammatory cytokines, i.e. TNF-α and IL-6, activate inflammation. The levels of IL-6 and TNF-α in raw 264.7 macrophage cells, evaluated by ELISA, increased following treatment with LPS (p value <0.01), while decreased in EF+LPS treated ones (p value <0.01) (Fig 3A and 3B). The transcript levels of IL-6 and TNF-α were analyzed by RT-PCR, they increased in LPS treated raw 264.7 macrophage cells, and reduced with treatment of EF in a dose-dependent manner (p value <0.01) (Fig 3C and 3D). We obtained further support for this observation using western blotting to detect the expression of IL-6 (p value <0.05, p value <0.01) and TNF-α (p value <0.01) (Fig 3E–3G).

### EF inhibited LPS-stimulated NF-КB activation in raw 264.7 macrophage cells

**Fig 4:**
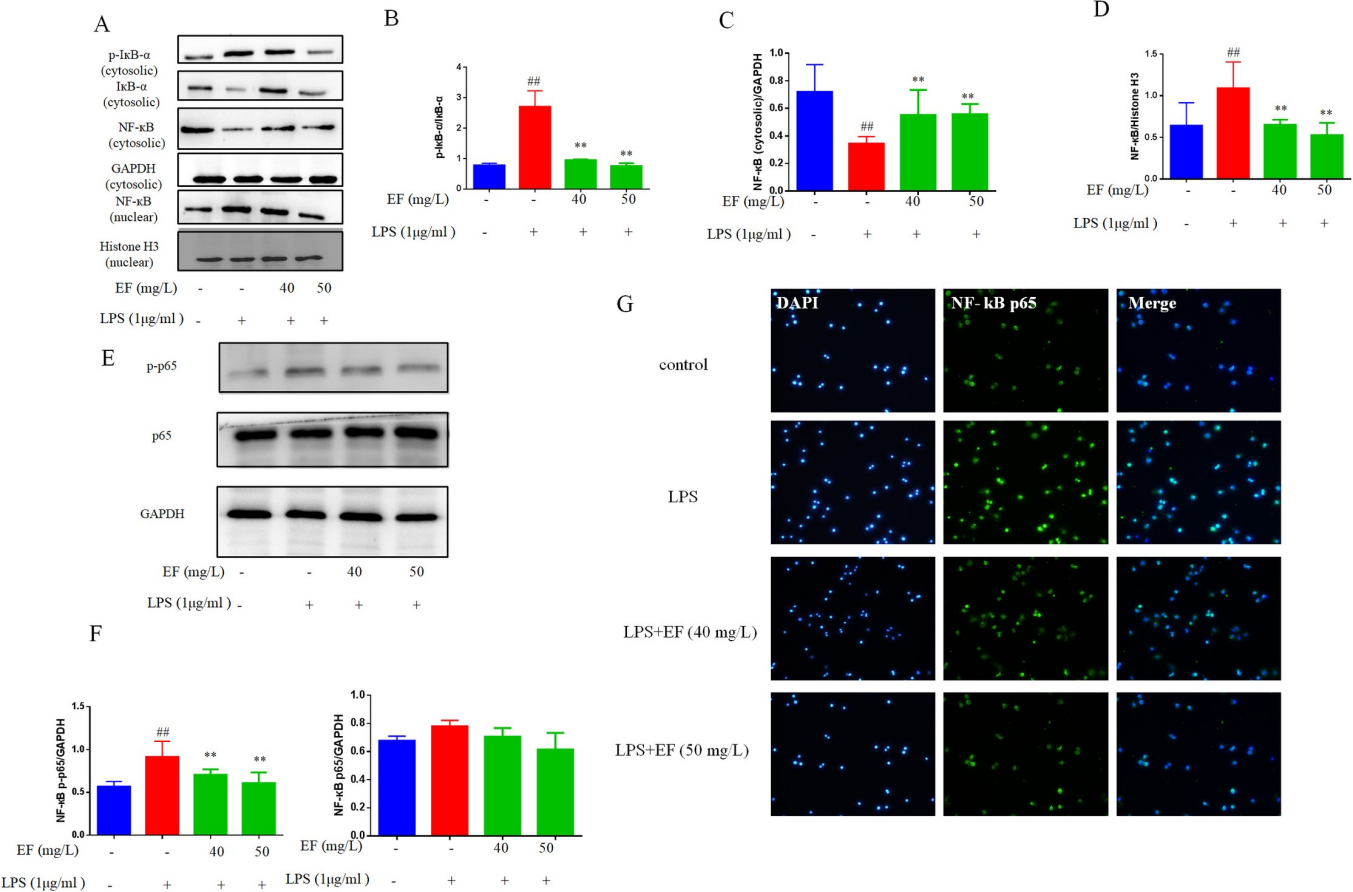
EF down-regulated LPS-stimulated NF-кB activation in RAW 264.7 macrophages cells. A/B/C/D/E/F: The protein levels of p-IкB-α, IкB-α, NF-кB p65 in cytosolic and NF-кB p65 in nuclear, and p- NF-кB p65 were determined by Western blotting analysis using specific antibodies. G: NF-кB p65 was determined using immunofluorescence analysis (50 μm). All data are shown as mean ± SEM (n = 6, per group) ##P<0.01 vs control; **P<0.01 vs LPS.

The expression levels of NF-КB-pathway-related protein were evaluated by western blotting. It was demonstrated that the degradation of IкBα increased in murine RAW264.7 macrophages following treatment with LPS. By contrast, the degradation of IкBα decreased in those treated with EF+LPS (p value <0.01) (Fig 4). The data showed that EF inhibited the translocation of NF-кB p65 from cytoplasm to nucleus in murine RAW264.7 macrophages treated with LPS (p value <0.01) (Fig 4).

### EF reduced LPS-induced oxidative stress

**Fig 5:**
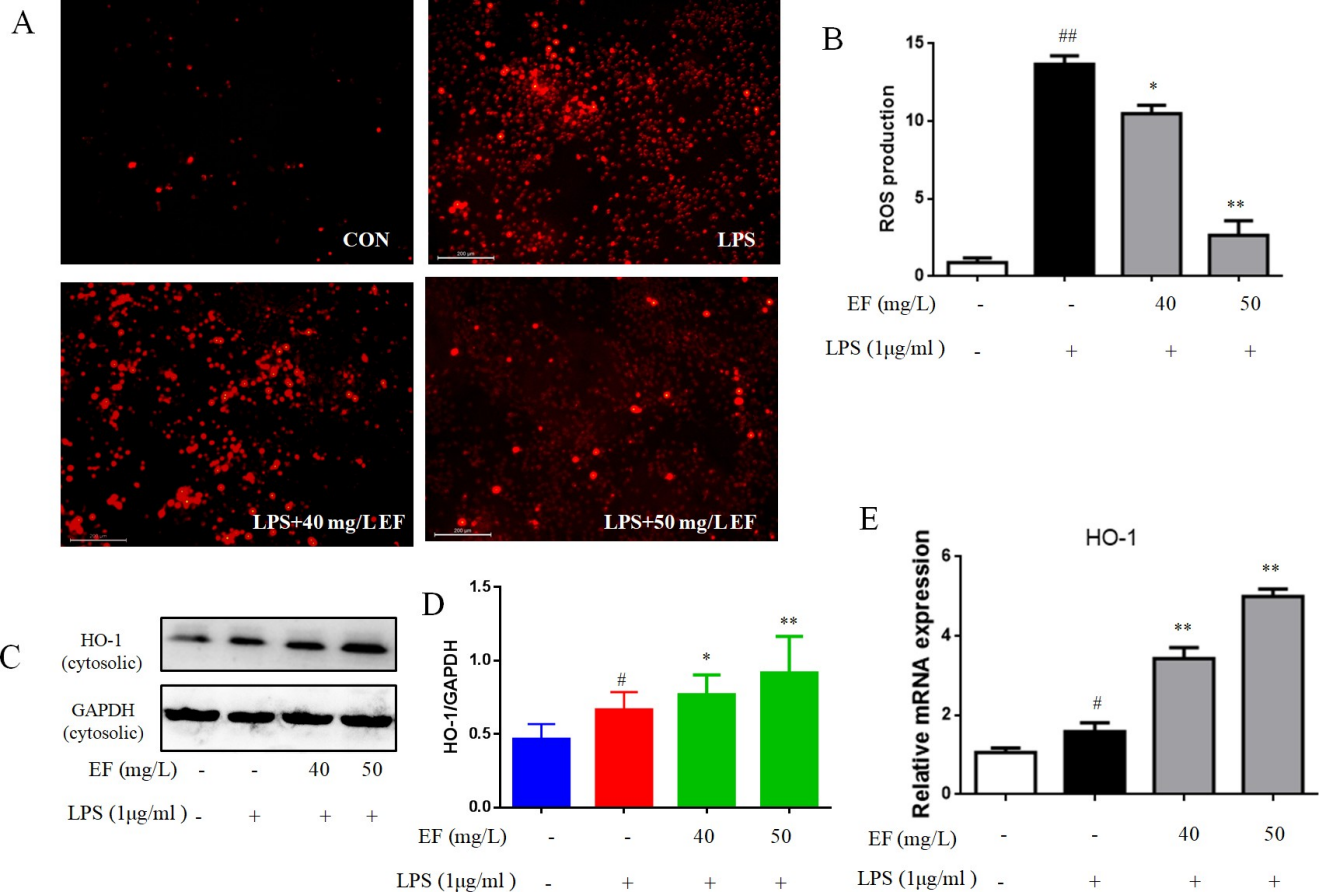
EF protected macrophages from oxidative stress. A/B: after stimulation, intracellular ROS levels were measured by DCFH-DA. C/D/E: The mRNA and protein levels of HO-1 were determined by Western blotting analysis using specific antibodies. All data are shown as mean ± SEM (n = 6, per group) #P<0.05, ##P<0.01 vs control; *P<0.05, **P<0.01 vs LPS.

**Fig 6:**
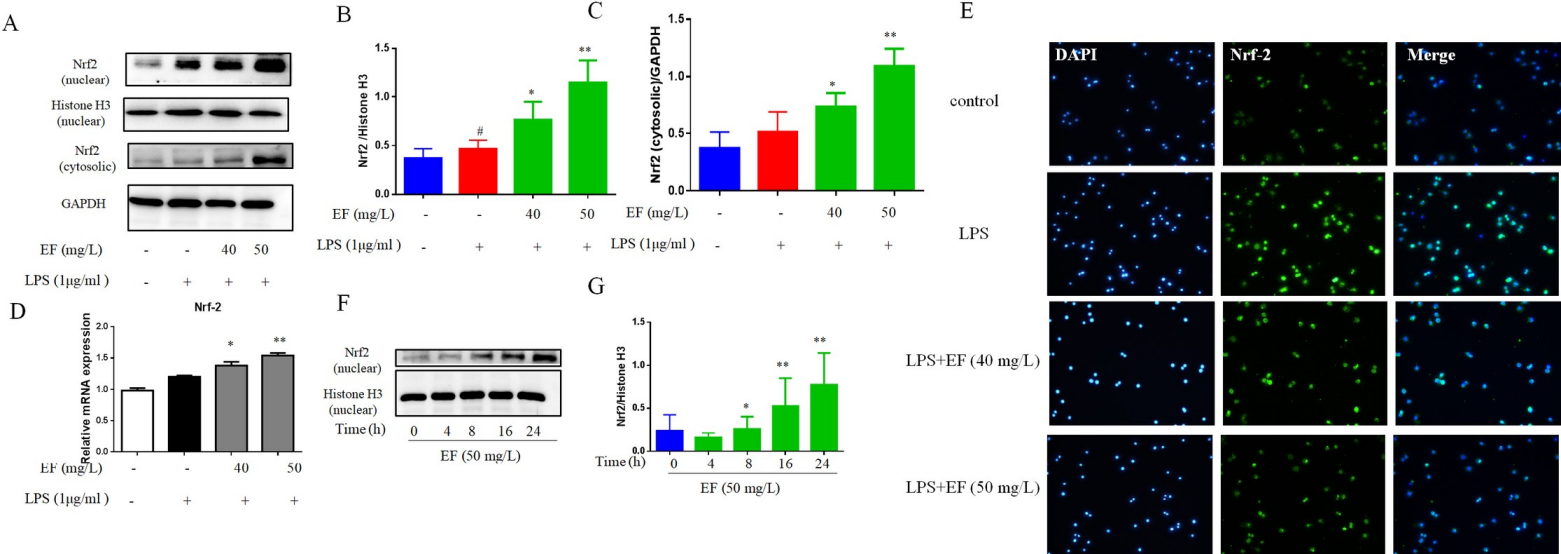
EF affects the expression of Nrf2. Effect of EF on the expression of Nrf2 in nuclear (A, B) and cytosolic (A, C). D: The mRNA levels of Nrf2 were determined by quantitative real-time PCR. E: Nrf2 was determined using immunofluorescence analysis (50 μm). Effect of the EF treatment time on the expression of Nrf2 (F, G) in the absence of LPS activated cells. All data are shown as mean ± SEM (n = 6, per group) #P<0.05, ##P<0.01 vs control; *P<0.05, **P<0.01 vs LPS.

LPS induces the oxidative stress, and then activates various inflammatory signaling pathways. We observed that LPS induced intracellular ROS levels, whereas EF treatment significantly reduced the oxidative stress (p value <0.05, p value <0.01) (Fig 5A and 5B). In our research, we determined whether EF induced the expression of HO1, which is a mediator of critical cellular responses against oxidative stress induced by toxicity and inflammatory responses. The transcript levels and protein expression of HO-1 were significantly enhanced by EF in contrast with LPS (p value <0.05, p value <0.01) (Fig 5C–5E). Nrf2 manifests the antioxidant responsive genes, which regulate the oxidative stress and maintain the cellular homeostasis by inducing SOD and stress-responsing protein HO-1 to catabolise superoxide ions [13]. We detected that up-regulator of HO-1, Nrf2, protein and transcript levels expression of Nrf2 were significantly increased by EF in contrast with LPS in both nuclear and cytoplasm (p value <0.05, p value <0.01) (Fig 6A–6D), the same with immunofluorescence data (Fig 6E). Furthermore, we found that EF significantly activated the expression of Nrf2 after 8 h in a time-dependent manner, as shown in Fig 6F and 6G EF attenuates inflammation by up-regulating the expression of Nrf2.

**Fig 7:**
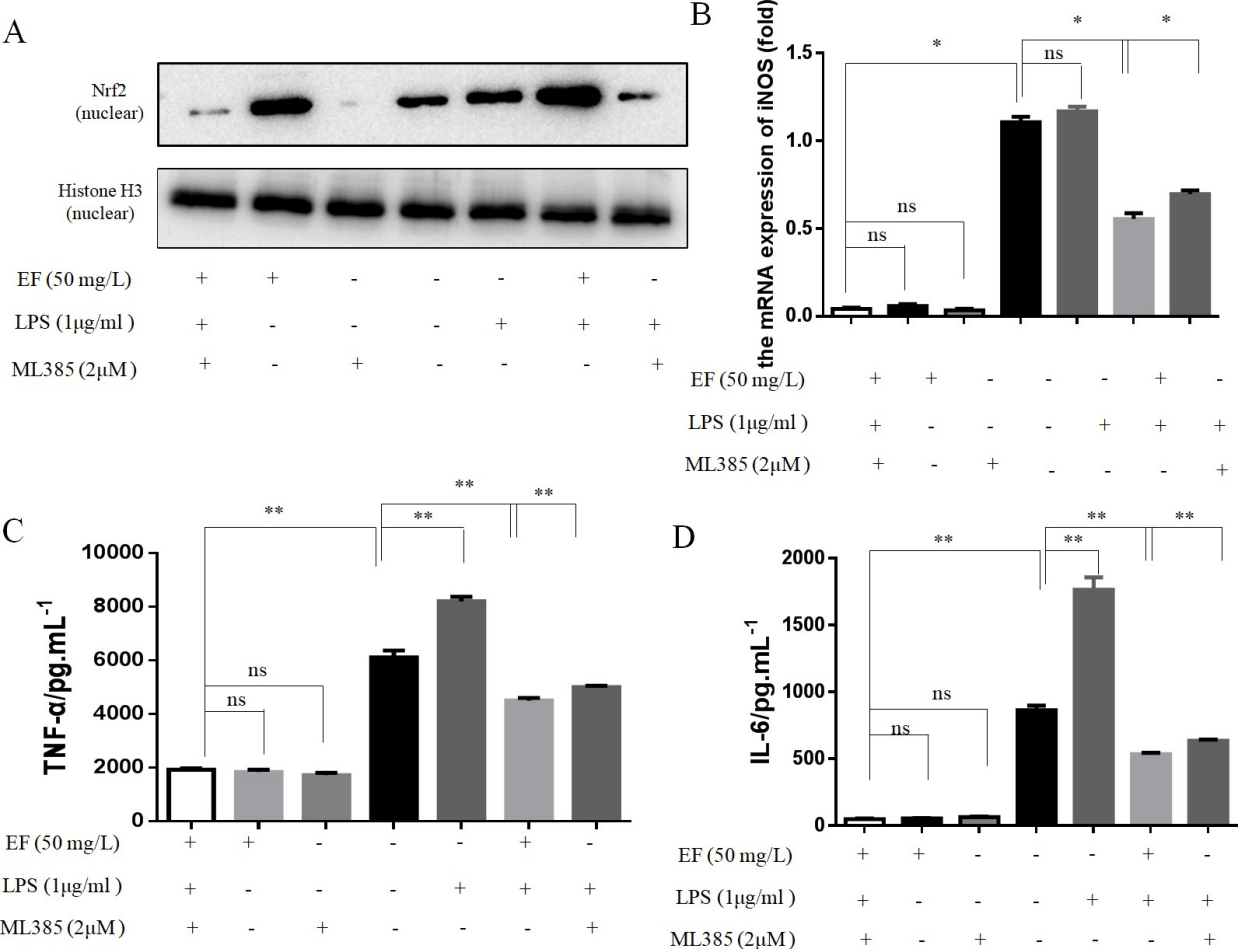
The inhibition of Nrf2 reversed the protective effect of EF on mRNA iNOS and production of TNF-α and IL-6. A: The protein levels of Nrf2 in nuclear were determined by Western blotting analysis using specific antibodies. B: The mRNA levels of iNOS were determined by quantitative real-time PCR. C/D: The supernatant levels of TNF-α and IL-6 were identified by ELISA.

**Fig 8:**
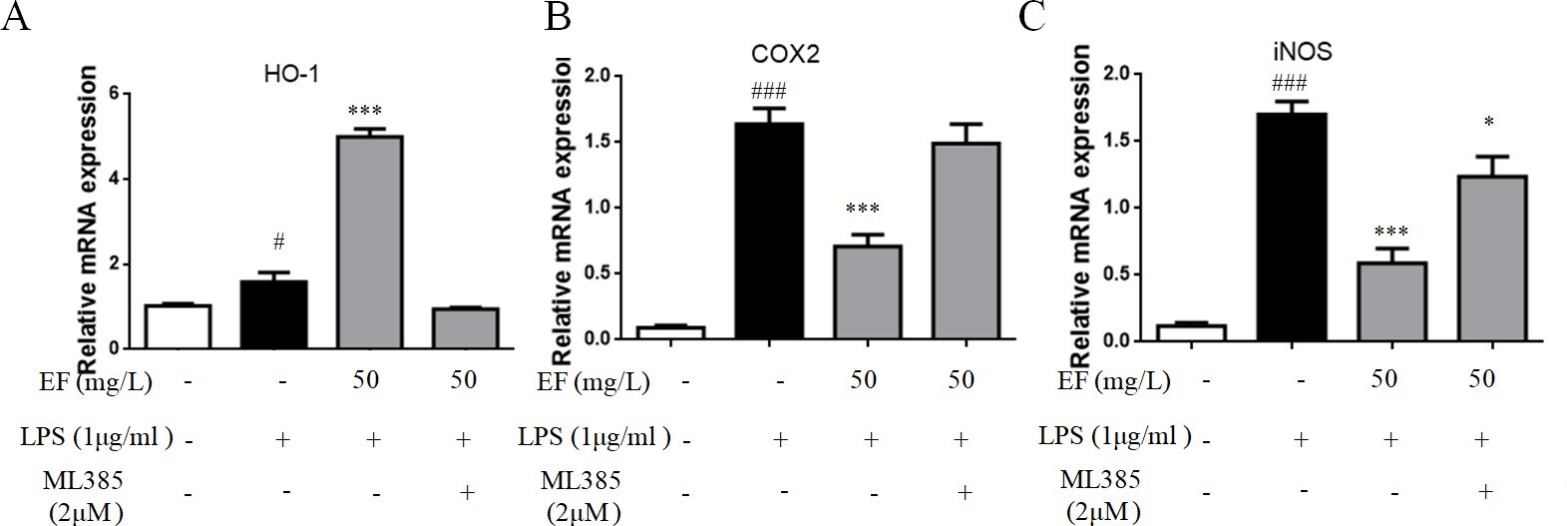
The inhibition of Nrf-2 reduced the mRNA of HO-1 and increased the mRNA of COX2 and iNOS. A/B/C: The mRNA levels of HO-1, iNOS and COX-2 were determined by quantitative real-time PCR. All data are shown as mean ± SEM (n = 6, per group) #P<0.05, ###P<0.001 vs control; *P<0.05, ***P<0.001 vs LPS.

To further verify that EF could activate Nrf2/ HO-1 pathway in raw 264.7 macrophage cells to inhibit inflammatory responses, the inhibitory effect of Nrf2 inhibitor ML385 (2μM) on mediatation of antioxidant activity by raw 264.7 macrophage cells was evaluated (Fig 7A). The transcript level of HO-1 was down-regulated and which of iNOS was up-regulated in raw 264.7 macrophage cells treated with ML385+LPS+EF (p value <0.05) (Figs 7B and 8A). As shown in Fig 7, both TNF-α and IL-6 were significantly enhanced after abolishing the expression of Nrf2, and co-treatment with ML385 and EF significantly reduced the LPS-induced TNF-α and IL-6 secretion (p value <0.01) and the transcript level of COX-2 and iNOS was up-regulated in raw 264.7 macrophage cells treated with ML385+LPS+EF (Fig 8B and 8C) (p value <0.05, p value <0.001).

### EF alleviated LPS-induced acute lung injury in mice

**Fig 9:**
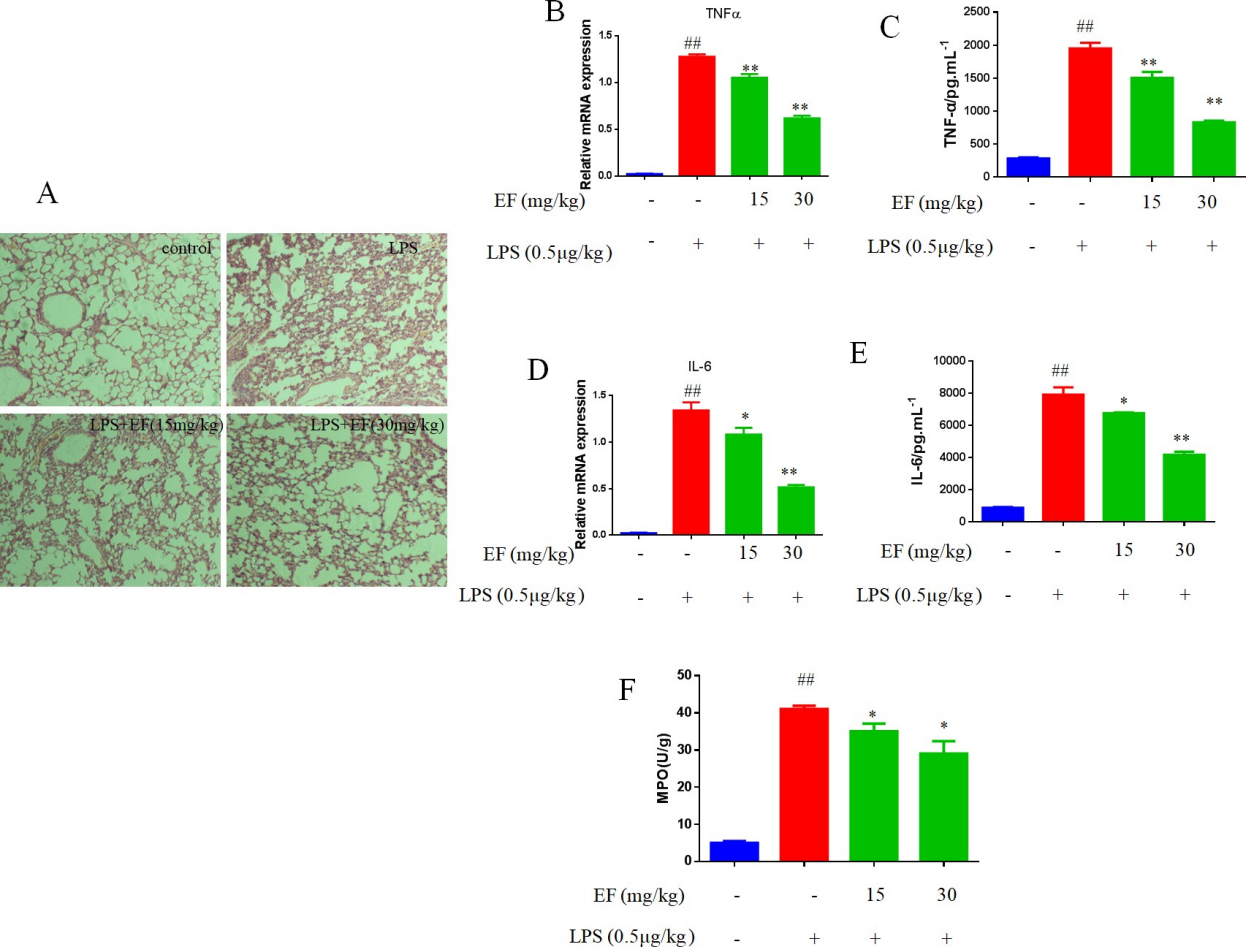
Effect of EF on LPS-induced acute lung injury in mice. A: lungs from different groups were processed for histological evaluation 8 h after the LPS. B/D: The mRNA levels of TNFα and IL-6 were determined by quantitative real-time PCR. C/E: The BALF levels of TNF-α, IL-6 and IL-1β, determined by ELISA. #P<0.05, ##P<0.01 vs control; *P<0.05, **P<0.01 vs LPS.

To confirm whether the anti-flammatory effects in vitro could be validated in vivo, we next examined the effect of EF in LPS-induced acute lung injury in mice. Pretreatment with EF significantly attenuated the degree of leukocyte infiltration (p value <0.05, p value <0.01) (Fig 9), reduced MPO activity, mRNA levels and secretion of TNF-α and IL-6.

## Discussion

**Fig 10:**
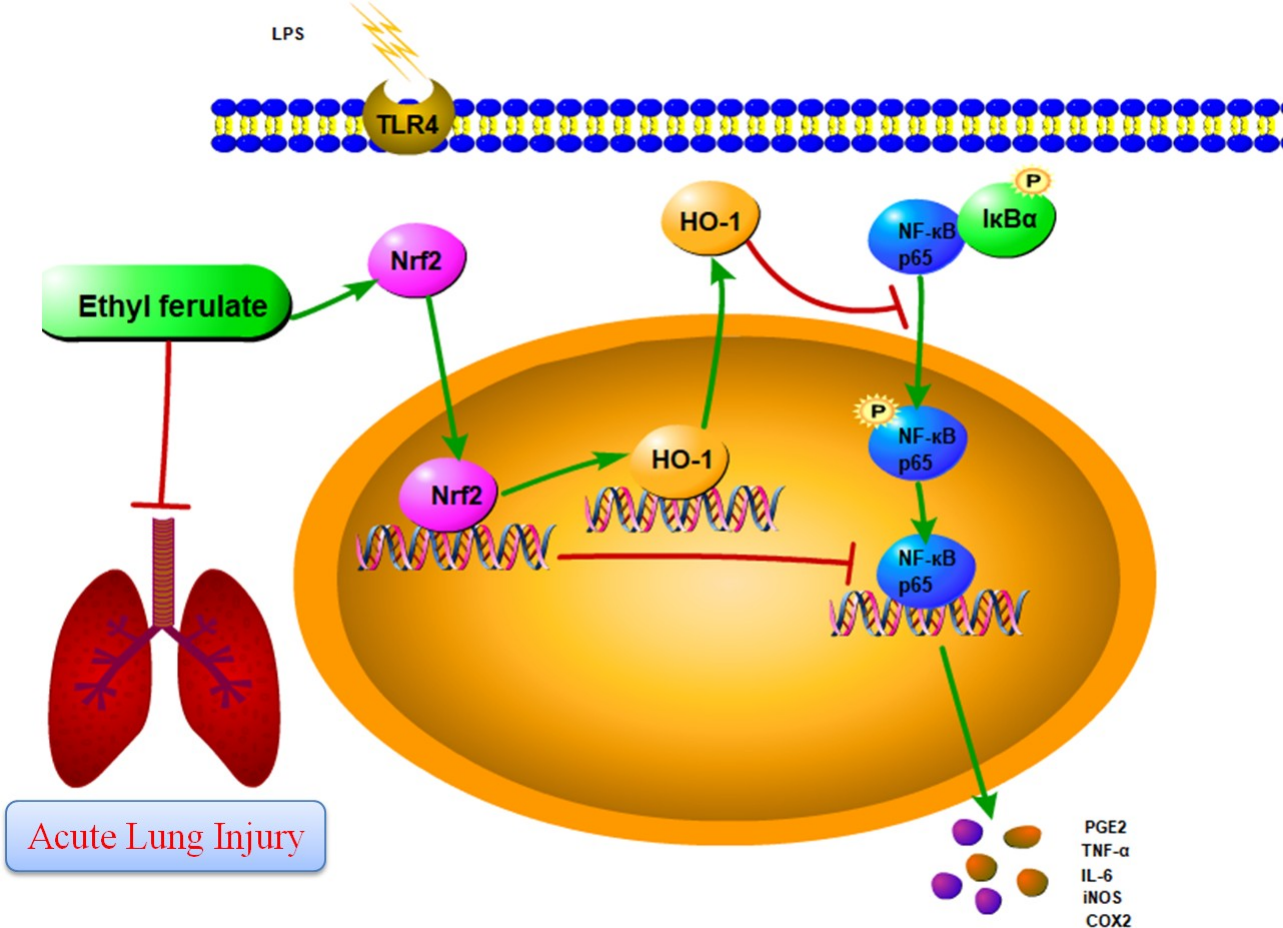
Schematic diagram of the mechanism. We systematically analyzed the mechanism of EF effect on LPS signaling, and found EF profoundly activated Nrf2/HO-1 signal transduction pathways and inhibited NF-кB signal pathways, known to be involved in the regulation of inflammatory responses, by Nrf2.

Some researches have demonstrated over-activation of macrophages plays an important role in the pathogenesis of acute and chronic inflammatory disorders. It could attenuate inflammatory disorders by inhibiting activation of macrophages [14, 15]. According to our study, we proved that EF could regulate the imbalance of LPS-induced inflammation by activating Nrf2/HO-1 pathway and blocking NF-кB pathway. The regulation of anti-inflammation by EF was achieved by activating Nrf2 (Fig 10).

EF has aroused interest due to anti-inflammatory and antioxidant. Yung-Chieh et al, reported pretreatment of FAEE (1 and 10 μm) in A10 cells could significantly leptin-induced proliferation and migration of aortic smooth muscle cells [16]. To investigate whether EF could attenuate oxidative stress in the retina, Masayuki et al, found EF (0.02 or 0.01 mM) did not affect the viability of ARPE-19 cells. Exposure to 0.4 mM H2O2 for 1 h caused a 50–60% loss in cell viability, and treatment with EF attenuated this cell damage [17]. According these researches, we chose different concentrations (10, 20, 40, 50, 80 mg/L) of EF and with or without LPS in murine RAW 264.7 macrophage cells. In this study, we found 1 μg/mL LPS have no effect on cell viability in raw 264.7 macrophage cells. while the concentrations 10, 20, 40, 50, 80 mg/L of EF no cellular toxicity in raw 264.7 macrophage cells stimulated by LPS.

LPS-stimulated macrophages could produce NO, which promotes inflammation [18]. The increase of inducible nitric oxide synthase (iNOS) is due to the induction of pro-inflammatory cytokines, including IL-6 and TNF-α. Our results indicated that EF inhibited the production of IL-6, TNF-α, IL-1βand iNOS in LPS-induced raw 264.7 macrophage cells. COX2 regulates inflammatory responses resulting in fever, pain, hypersensitivity and edema, and is an important factor for synthesizing PGs [19], which is a key mediator of inflammation and contributes to hypersensitivity to pain. In order to comprehensively learn the anti-inflammatory activity of EF, the effect of EF on production of COX2 and PGE2 was detected. Our results showed that EF inhibited the expression of COX2 and PGE2, reduced inflammatory cytokines and alleviated the development of inflammation.

As a transcription factor, NF-кB plays a vital role in numerous processes, including inflammation, immunoreaction and cell proliferation. NF-кB is an important signaling pathway in the development of various inflammation-mediated diseases [20], also a upstream regulatory factor of pro-inflammatory cytokines, including IL-6 and TNF-α. In this study, we found EF decreased NF-кB transportation to nucleus by down-regulating NF-кB p65 in nucleus and increase it in cytoplasm. Besides, the expression of p-IкBα was down-regulated in cytoplasm, also suggesting that decreased NF-кB was freed to enter the nucleus.

As signaling molecules, ROS triggered many biological responses upon exposure to the stressful environments. Although moderate level of ROS is required for the removal of pathogens, overproduction of ROS is thought to be harmful in inflammatory diseases [21]. We found the production of ROS in raw 264.7 macrophage cells with LPS increased, which could be inhibited by EF. As the transcription factor essential for protection against oxidative injury, Nrf2 could regulate the expression of HO-1 [22] with the function of anti-inflammation and inhibition of the nuclear translocation of NF-кB [23]. In this research, Nrf2/HO-1 signaling pathway could be enhanced in raw 264.7 macrophage cells with LPS+EF, which is beneficial to the treatment of EF. To investigate whether the treatment of EF depends on Nrf2, the inhibition of Nrf2- ML385 was used in cells. The results showed that ML385 significantly up-regulated the expression of NF-кB in nuclear, increased the transcript levels of COX-2 and iNOS, and decreased that of HO-1.

We also analyzed the effect of EF on LPS-induced acute lung injury in mice. In our study, we found the pretreatment of mice with EF could prevent LPS-induced lung histopathological changes and generation of pro-inflammatory cytokines, and decrease MPO activity.

Present study provided evidences to demonstrate that EF possessed potent anti-inflammatory effect by inhibiting pro-inflammatory cytokines secretion in RAW 264.7 macrophages and in mice. Furthermore, we, for the first time, systematically analyzed the mechanism of EF effect on LPS signaling, and found EF profoundly activated Nrf2/HO-1 signal transduction pathways and inhibited NF-кB signal pathways, known to be involved in the regulation of inflammatory responses, by Nrf2 in RAW 264.7 macrophages. Future studies, we will analyze possible mechanism of anti-inflammation effect of EF in mice and the suitable concentration on EF on human body.

## Supporting information

S1 File Western blot. (ZIP)
