# Correction: Ethyl ferulate contributes to the inhibition of the inflammatory responses in murine RAW 264.7 macrophage cells and acute lung injury in mice

**DOI:** 10.1371/journal.pone.0270447

**Published:** 2022-06-21

**Authors:** Yu Wang, Xuan Zhang, Linger Li, Zhao Zhang, Chengxi Wei, Guohua Gong

In the Funding statement, the grant number from the funder Outstanding Youth of the natural science foundation of Inner Mongolia is listed incorrectly. The correct grant number is: No.2018JQ01.
