# Supplementary figures and images for: Ethyl ferulate contributes to the inhibition of the inflammatory responses in murine RAW 264.7 macrophage cells and acute lung injury in mice
Source: PLoS One. 2021 May 26;16(5):e0251578. doi: 10.1371/journal.pone.0251578 (PMC8153479; doi:10.1371/journal.pone.0251578)

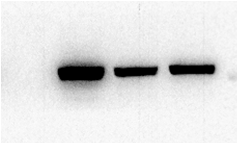

Supplement: S1 File — (ZIP) [file pone.0251578.s001.zip › Western blot/Fig2 cox2-1.tif]

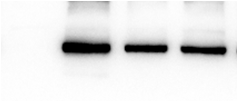

Supplement: S1 File — (ZIP) [file pone.0251578.s001.zip › Western blot/Fig2 cox2-2.tif]

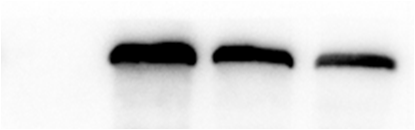

Supplement: S1 File — (ZIP) [file pone.0251578.s001.zip › Western blot/Fig2 cox2-3.tif]

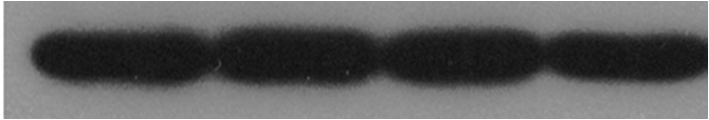

Supplement: S1 File — (ZIP) [file pone.0251578.s001.zip › Western blot/Fig2 gapdh-1.tif]

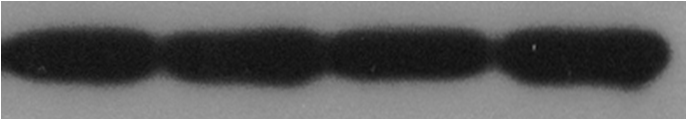

Supplement: S1 File — (ZIP) [file pone.0251578.s001.zip › Western blot/Fig2 gapdh-2.tif]

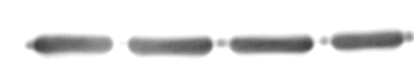

Supplement: S1 File — (ZIP) [file pone.0251578.s001.zip › Western blot/Fig2 gapdh-3.tif]

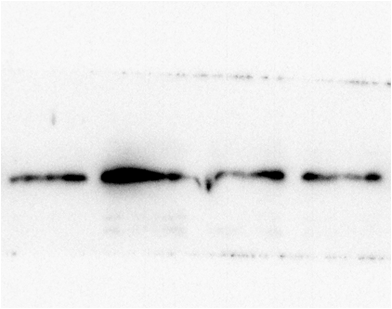

Supplement: S1 File — (ZIP) [file pone.0251578.s001.zip › Western blot/Fig2 iNOs-1.tif]

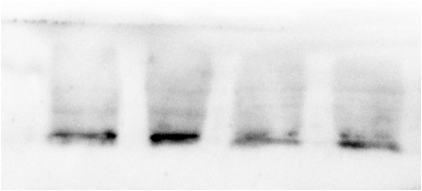

Supplement: S1 File — (ZIP) [file pone.0251578.s001.zip › Western blot/Fig2 iNOs-2.tif]

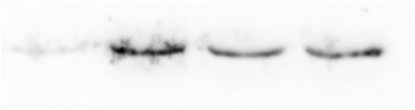

Supplement: S1 File — (ZIP) [file pone.0251578.s001.zip › Western blot/Fig2 iNOs-3.tif]

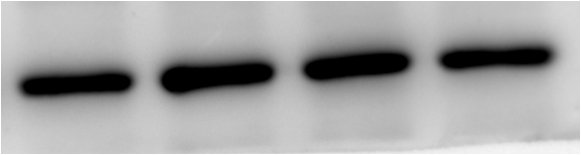

Supplement: S1 File — (ZIP) [file pone.0251578.s001.zip › Western blot/Fig3 gapdh-1.tif]

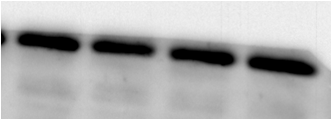

Supplement: S1 File — (ZIP) [file pone.0251578.s001.zip › Western blot/Fig3 gapdh-2.tif]

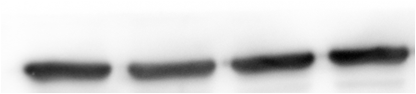

Supplement: S1 File — (ZIP) [file pone.0251578.s001.zip › Western blot/Fig3 gapdh-3.tif]

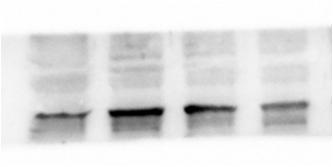

Supplement: S1 File — (ZIP) [file pone.0251578.s001.zip › Western blot/Fig3 IL6-1.tif]

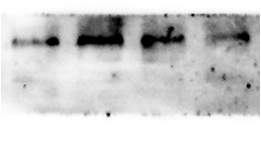

Supplement: S1 File — (ZIP) [file pone.0251578.s001.zip › Western blot/Fig3 IL6-2.tif]

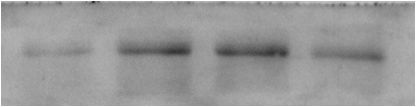

Supplement: S1 File — (ZIP) [file pone.0251578.s001.zip › Western blot/Fig3 IL6-3.tif]

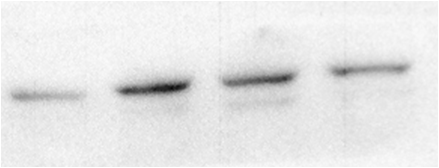

Supplement: S1 File — (ZIP) [file pone.0251578.s001.zip › Western blot/Fig3 tnfa-1.tif]

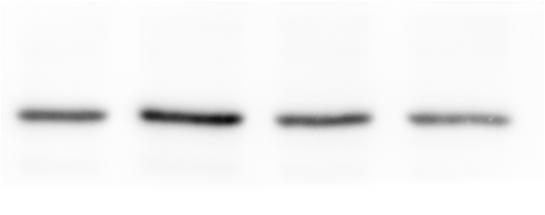

Supplement: S1 File — (ZIP) [file pone.0251578.s001.zip › Western blot/Fig3 tnfa-2.tif]

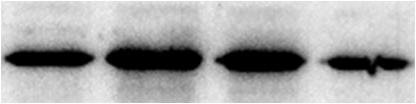

Supplement: S1 File — (ZIP) [file pone.0251578.s001.zip › Western blot/Fig3 tnfa-3.tif]

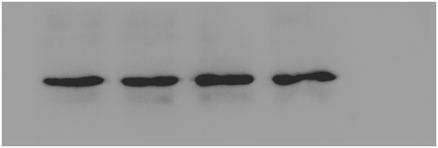

Supplement: S1 File — (ZIP) [file pone.0251578.s001.zip › Western blot/Fig4 histone H3 (nuclear)-1.tif]

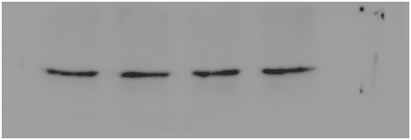

Supplement: S1 File — (ZIP) [file pone.0251578.s001.zip › Western blot/Fig4 histone H3 (nuclear)-2.tif]

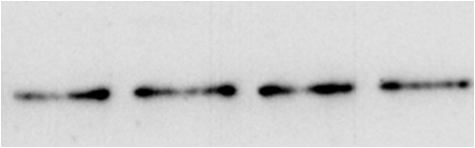

Supplement: S1 File — (ZIP) [file pone.0251578.s001.zip › Western blot/Fig4 histone H3 (nuclear)-3.tif]

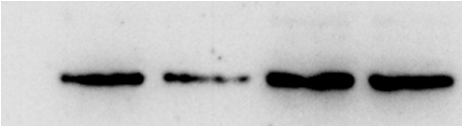

Supplement: S1 File — (ZIP) [file pone.0251578.s001.zip › Western blot/Fig4 IкB-α (cytosolic)-1.tif]

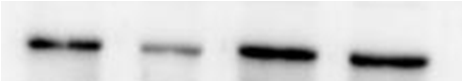

Supplement: S1 File — (ZIP) [file pone.0251578.s001.zip › Western blot/Fig4 IкB-α (cytosolic)-2.tif]

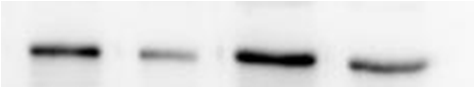

Supplement: S1 File — (ZIP) [file pone.0251578.s001.zip › Western blot/Fig4 IкB-α (cytosolic)-3.tif]

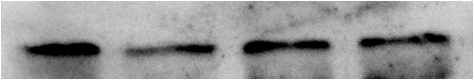

Supplement: S1 File — (ZIP) [file pone.0251578.s001.zip › Western blot/Fig4 NF-кB (cytosolic)-1.tif]

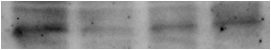

Supplement: S1 File — (ZIP) [file pone.0251578.s001.zip › Western blot/Fig4 NF-кB (cytosolic)-2.tif]

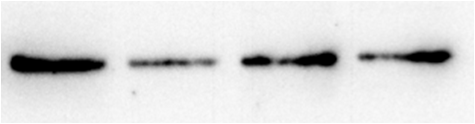

Supplement: S1 File — (ZIP) [file pone.0251578.s001.zip › Western blot/Fig4 NF-кB (cytosolic)-3.tif]

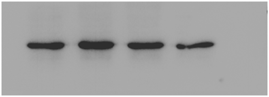

Supplement: S1 File — (ZIP) [file pone.0251578.s001.zip › Western blot/Fig4 NF-кB (nuclear)-1.tif]

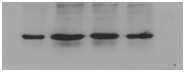

Supplement: S1 File — (ZIP) [file pone.0251578.s001.zip › Western blot/Fig4 NF-кB (nuclear)-2.tif]

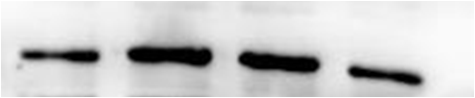

Supplement: S1 File — (ZIP) [file pone.0251578.s001.zip › Western blot/Fig4 NF-кB (nuclear)-3.tif]

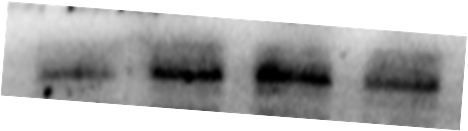

Supplement: S1 File — (ZIP) [file pone.0251578.s001.zip › Western blot/Fig4 p-IкB-α (cytosolic)-1.tif]

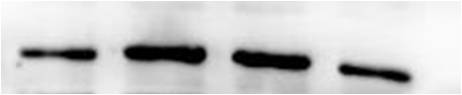

Supplement: S1 File — (ZIP) [file pone.0251578.s001.zip › Western blot/Fig4 p-IкB-α (cytosolic)-2.tif.tif]

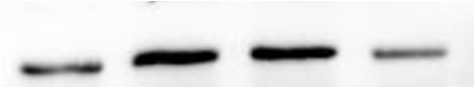

Supplement: S1 File — (ZIP) [file pone.0251578.s001.zip › Western blot/Fig4 p-IкB-α (cytosolic)-3.tif]

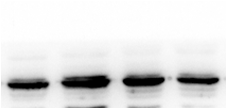

Supplement: S1 File — (ZIP) [file pone.0251578.s001.zip › Western blot/Fig4 p-p65-1.tif]

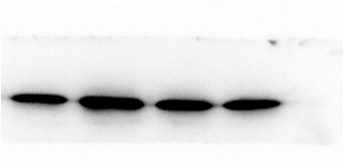

Supplement: S1 File — (ZIP) [file pone.0251578.s001.zip › Western blot/Fig4 p-p65-2.tif]

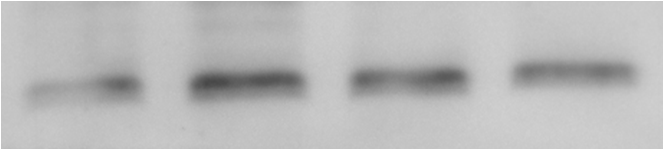

Supplement: S1 File — (ZIP) [file pone.0251578.s001.zip › Western blot/Fig4 p-p65-3.tif]

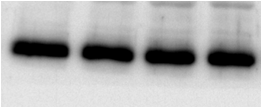

Supplement: S1 File — (ZIP) [file pone.0251578.s001.zip › Western blot/Fig4 p65-1.tif]

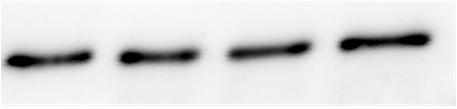

Supplement: S1 File — (ZIP) [file pone.0251578.s001.zip › Western blot/Fig4 p65-2.tif]

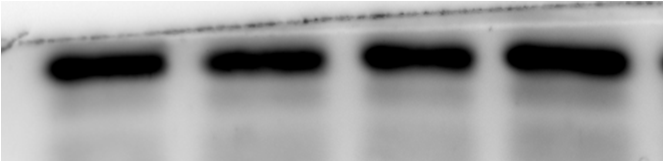

Supplement: S1 File — (ZIP) [file pone.0251578.s001.zip › Western blot/Fig4 p65-3.tif]

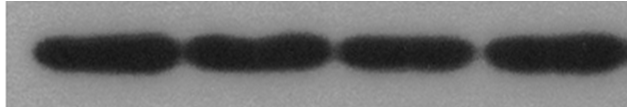

Supplement: S1 File — (ZIP) [file pone.0251578.s001.zip › Western blot/Fig4a gapdh-1.tif]

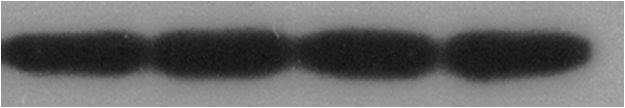

Supplement: S1 File — (ZIP) [file pone.0251578.s001.zip › Western blot/Fig4a gapdh-2.tif]

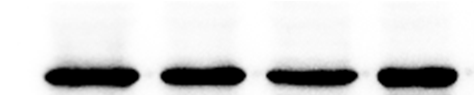

Supplement: S1 File — (ZIP) [file pone.0251578.s001.zip › Western blot/Fig4a gapdh-3.tif]

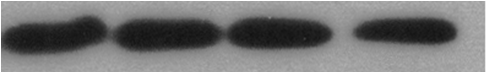

Supplement: S1 File — (ZIP) [file pone.0251578.s001.zip › Western blot/Fig4e GAPDH-2.tif]

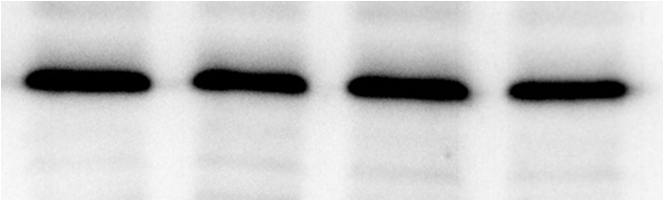

Supplement: S1 File — (ZIP) [file pone.0251578.s001.zip › Western blot/Fig4e GAPDH-3.tif]

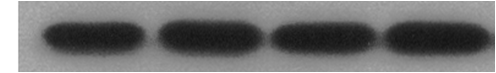

Supplement: S1 File — (ZIP) [file pone.0251578.s001.zip › Western blot/Fig4e GAPDH1-.tif]

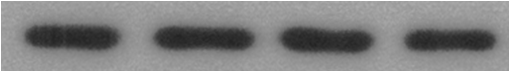

Supplement: S1 File — (ZIP) [file pone.0251578.s001.zip › Western blot/Fig5 GAPDH (cytosolic)-1.tif]

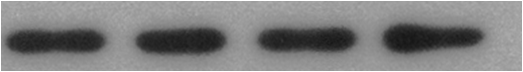

Supplement: S1 File — (ZIP) [file pone.0251578.s001.zip › Western blot/Fig5 GAPDH (cytosolic)-2.tif]

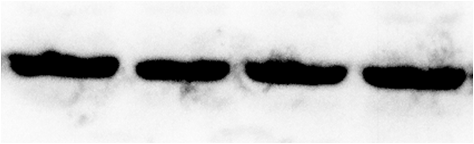

Supplement: S1 File — (ZIP) [file pone.0251578.s001.zip › Western blot/Fig5 GAPDH (cytosolic)-3.tif]

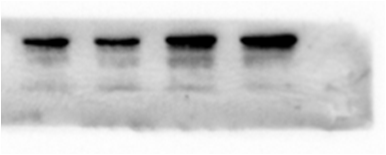

Supplement: S1 File — (ZIP) [file pone.0251578.s001.zip › Western blot/Fig5 ho-1 (cytosolic)-1.tif]

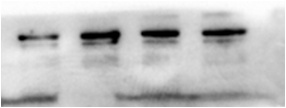

Supplement: S1 File — (ZIP) [file pone.0251578.s001.zip › Western blot/Fig5 ho-1 (cytosolic)-2.tif]

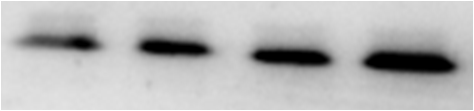

Supplement: S1 File — (ZIP) [file pone.0251578.s001.zip › Western blot/Fig5 ho-1 (cytosolic)-3.tif]

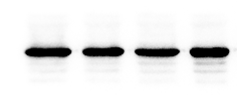

Supplement: S1 File — (ZIP) [file pone.0251578.s001.zip › Western blot/Fig6A GAPDH-1.tif]

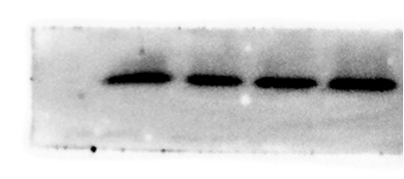

Supplement: S1 File — (ZIP) [file pone.0251578.s001.zip › Western blot/Fig6A GAPDH-2.tif]

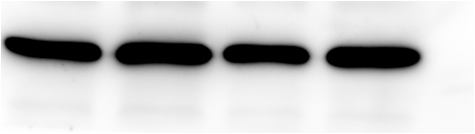

Supplement: S1 File — (ZIP) [file pone.0251578.s001.zip › Western blot/Fig6A GAPDH-3.tif]

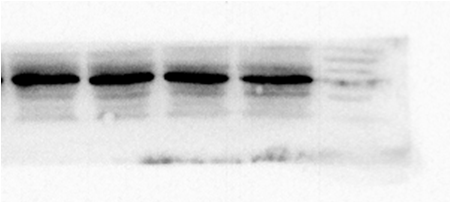

Supplement: S1 File — (ZIP) [file pone.0251578.s001.zip › Western blot/Fig6A histone H3-1.tif]

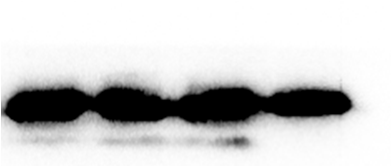

Supplement: S1 File — (ZIP) [file pone.0251578.s001.zip › Western blot/Fig6A histone H3-2.tif]

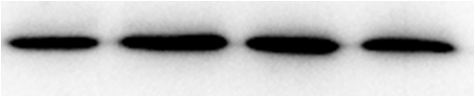

Supplement: S1 File — (ZIP) [file pone.0251578.s001.zip › Western blot/Fig6A histone H3-3.tif]

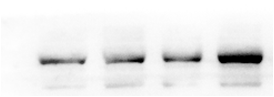

Supplement: S1 File — (ZIP) [file pone.0251578.s001.zip › Western blot/Fig6A nrf2(cytosolic)-1.tif]

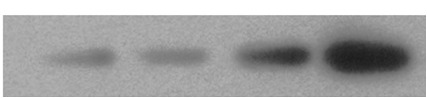

Supplement: S1 File — (ZIP) [file pone.0251578.s001.zip › Western blot/Fig6A nrf2(cytosolic)-2.tif]

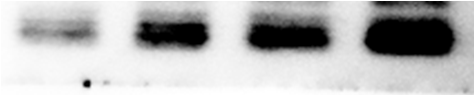

Supplement: S1 File — (ZIP) [file pone.0251578.s001.zip › Western blot/Fig6A nrf2(cytosolic)-3.tif]

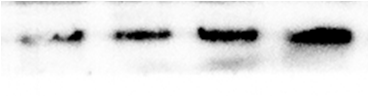

Supplement: S1 File — (ZIP) [file pone.0251578.s001.zip › Western blot/Fig6A nrf2(nuclear)-1.tif]

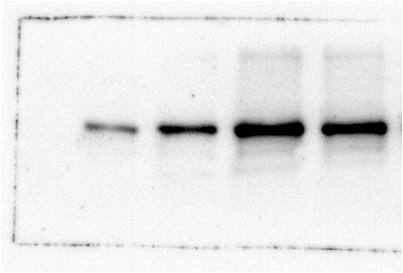

Supplement: S1 File — (ZIP) [file pone.0251578.s001.zip › Western blot/Fig6A nrf2(nuclear)-2.tif]

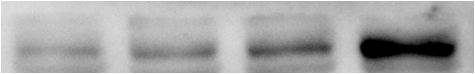

Supplement: S1 File — (ZIP) [file pone.0251578.s001.zip › Western blot/Fig6A nrf2(nuclear)-3.tif]

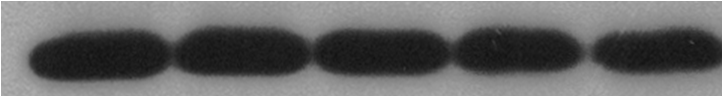

Supplement: S1 File — (ZIP) [file pone.0251578.s001.zip › Western blot/Fig6F histone H3-1.tif]

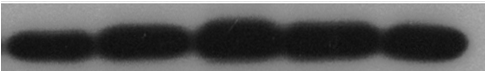

Supplement: S1 File — (ZIP) [file pone.0251578.s001.zip › Western blot/Fig6F histone H3-2.tif]

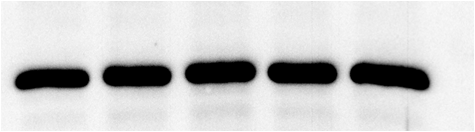

Supplement: S1 File — (ZIP) [file pone.0251578.s001.zip › Western blot/Fig6F histone H3-3.tif]

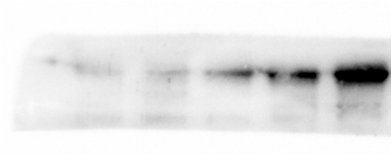

Supplement: S1 File — (ZIP) [file pone.0251578.s001.zip › Western blot/Fig6F nrf2(nuclear)-1.tif]

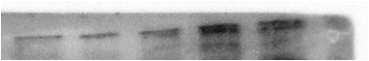

Supplement: S1 File — (ZIP) [file pone.0251578.s001.zip › Western blot/Fig6F nrf2(nuclear)-2.tif]

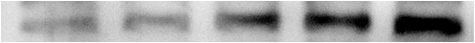

Supplement: S1 File — (ZIP) [file pone.0251578.s001.zip › Western blot/Fig6F nrf2(nuclear)-3.tif]

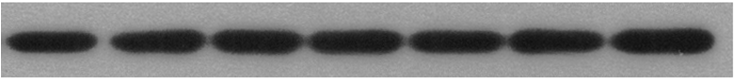

Supplement: S1 File — (ZIP) [file pone.0251578.s001.zip › Western blot/Fig7 histone H3 (nuclear)-1.tif]

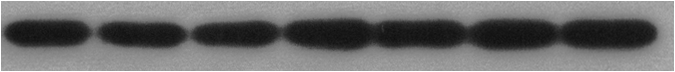

Supplement: S1 File — (ZIP) [file pone.0251578.s001.zip › Western blot/Fig7 histone H3 (nuclear)-2.tif]

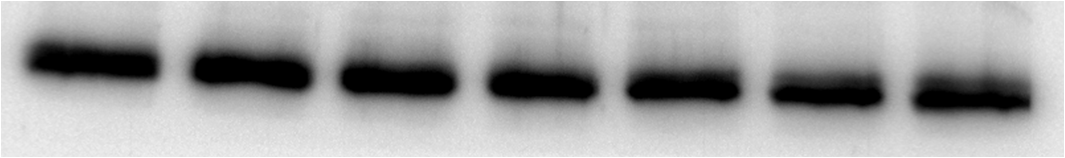

Supplement: S1 File — (ZIP) [file pone.0251578.s001.zip › Western blot/Fig7 histone H3 (nuclear)-3.tif]

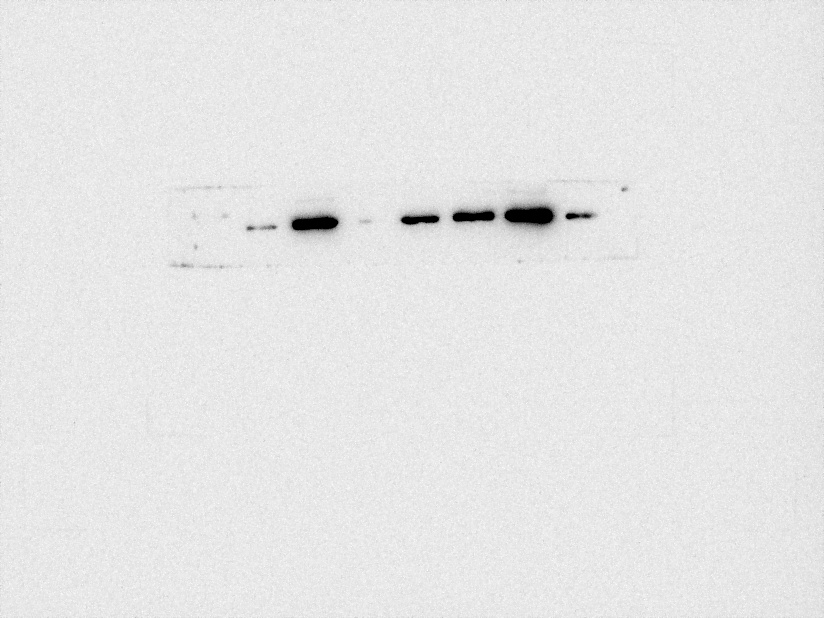

Supplement: S1 File — (ZIP) [file pone.0251578.s001.zip › Western blot/Fig7 nrf2 (nuclear)-1.tif]

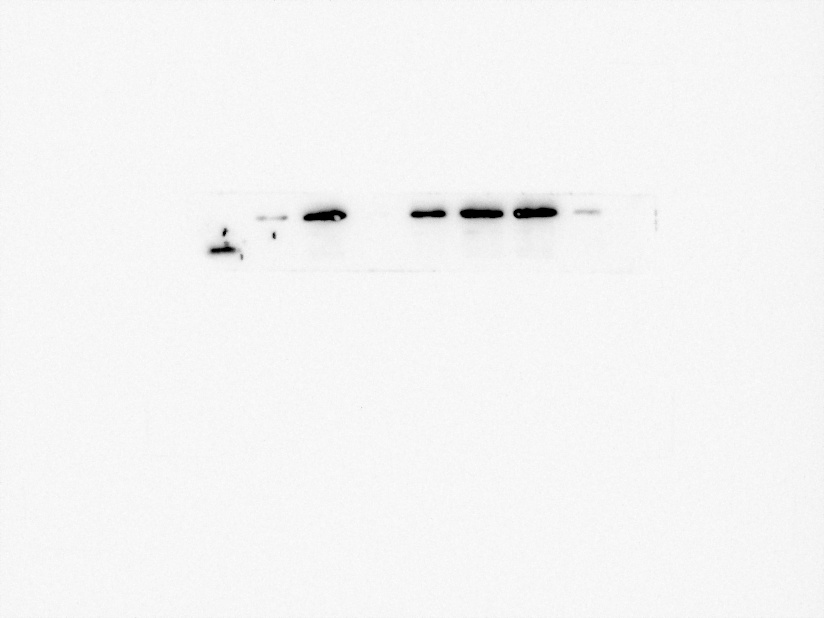

Supplement: S1 File — (ZIP) [file pone.0251578.s001.zip › Western blot/Fig7 nrf2 (nuclear)-2.tif]

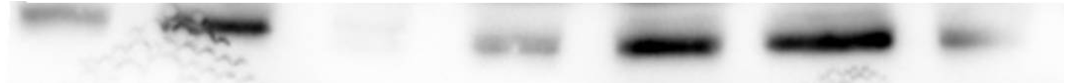

Supplement: S1 File — (ZIP) [file pone.0251578.s001.zip › Western blot/Fig7 nrf2 (nuclear)-3.tif]
