# Supplementary material for: Ethyl ferulate contributes to the inhibition of the inflammatory responses in murine RAW 264.7 macrophage cells and acute lung injury in mice
Source: PLoS One. 2021 May 26;16(5):e0251578. doi: 10.1371/journal.pone.0251578 (PMC8153479; doi:10.1371/journal.pone.0251578)
Supplement: Peer review history

# Peer review: Decision Letter 0

**DOI:** 10.1371/journal.pone.0251578.r001

**Authors:** Yi Cao

12 Apr 2021

PONE-D-21-04411

Ethyl Ferulate Contributes to the Inhibition of the Inflammatory Responses in Murine RAW 264.7 Macrophage Cells and Acute Lung Injury in Mice

PLOS ONE

Dear Dr. gong,

Thank you for submitting your manuscript to PLOS ONE. After careful consideration, we feel that it has merit but does not fully meet PLOS ONE’s publication criteria as it currently stands. Therefore, we invite you to submit a revised version of the manuscript that addresses the points raised during the review process.

Please submit your revised manuscript by May 24 2021 11:59PM. If you will need more time than this to complete your revisions, please reply to this message or contact the journal office at plosone@plos.org. When you're ready to submit your revision, log on to https://www.editorialmanager.com/pone/ and select the 'Submissions Needing Revision' folder to locate your manuscript file.

Please include the following items when submitting your revised manuscript:

- A rebuttal letter that responds to each point raised by the academic editor and reviewer(s). You should upload this letter as a separate file labeled 'Response to Reviewers'.
- A marked-up copy of your manuscript that highlights changes made to the original version. You should upload this as a separate file labeled 'Revised Manuscript with Track Changes'.
- An unmarked version of your revised paper without tracked changes. You should upload this as a separate file labeled 'Manuscript'.

If you would like to make changes to your financial disclosure, please include your updated statement in your cover letter. Guidelines for resubmitting your figure files are available below the reviewer comments at the end of this letter.

If applicable, we recommend that you deposit your laboratory protocols in protocols.io to enhance the reproducibility of your results. Protocols.io assigns your protocol its own identifier (DOI) so that it can be cited independently in the future. For instructions see: http://journals.plos.org/plosone/s/submission-guidelines#loc-laboratory-protocols. Additionally, PLOS ONE offers an option for publishing peer-reviewed Lab Protocol articles, which describe protocols hosted on protocols.io. Read more information on sharing protocols at https://plos.org/protocols?utm_medium=editorial-email&utm_source=authorletters&utm_campaign=protocols.

We look forward to receiving your revised manuscript.

Kind regards,

Yi Cao

Academic Editor

PLOS ONE

Journal Requirements:

When submitting your revision, we need you to address these additional requirements.

1. Please ensure that your manuscript meets PLOS ONE's style requirements, including those for file naming. The PLOS ONE style templates can be found at

https://journals.plos.org/plosone/s/file?id=wjVg/PLOSOne_formatting_sample_main_body.pdf and

https://journals.plos.org/plosone/s/file?id=ba62/PLOSOne_formatting_sample_title_authors_affiliations.pdf

2. PLOS ONE now requires that authors provide the original uncropped and unadjusted images underlying all blot or gel results reported in a submission’s figures or Supporting Information files. This policy and the journal’s other requirements for blot/gel reporting and figure preparation are described in detail at https://journals.plos.org/plosone/s/figures#loc-blot-and-gel-reporting-requirements and https://journals.plos.org/plosone/s/figures#loc-preparing-figures-from-image-files. When you submit your revised manuscript, please ensure that your figures adhere fully to these guidelines and provide the original underlying images for all blot or gel data reported in your submission. See the following link for instructions on providing the original image data: https://journals.plos.org/plosone/s/figures#loc-original-images-for-blots-and-gels.

In your cover letter, please note whether your blot/gel image data are in Supporting Information or posted at a public data repository, provide the repository URL if relevant, and provide specific details as to which raw blot/gel images, if any, are not available. Email us at plosone@plos.org if you have any questions.

3. PLOS requires an ORCID iD for the corresponding author in Editorial Manager on papers submitted after December 6th, 2016. Please ensure that you have an ORCID iD and that it is validated in Editorial Manager. To do this, go to ‘Update my Information’ (in the upper left-hand corner of the main menu), and click on the Fetch/Validate link next to the ORCID field. This will take you to the ORCID site and allow you to create a new iD or authenticate a pre-existing iD in Editorial Manager. Please see the following video for instructions on linking an ORCID iD to your Editorial Manager account: https://www.youtube.com/watch?v=_xcclfuvtxQ

[Note: HTML markup is below. Please do not edit.]

Reviewers' comments:

Reviewer's Responses to Questions

**Comments to the Author**

1. Is the manuscript technically sound, and do the data support the conclusions?

The manuscript must describe a technically sound piece of scientific research with data that supports the conclusions. Experiments must have been conducted rigorously, with appropriate controls, replication, and sample sizes. The conclusions must be drawn appropriately based on the data presented. 

Reviewer #1: Yes

Reviewer #2: Yes

**********

2. Has the statistical analysis been performed appropriately and rigorously? 

Reviewer #1: I Don't Know

Reviewer #2: Yes

**********

3. Have the authors made all data underlying the findings in their manuscript fully available?

The PLOS Data policy requires authors to make all data underlying the findings described in their manuscript fully available without restriction, with rare exception (please refer to the Data Availability Statement in the manuscript PDF file). The data should be provided as part of the manuscript or its supporting information, or deposited to a public repository. For example, in addition to summary statistics, the data points behind means, medians and variance measures should be available. If there are restrictions on publicly sharing data—e.g. participant privacy or use of data from a third party—those must be specified.

Reviewer #1: Yes

Reviewer #2: Yes

**********

4. Is the manuscript presented in an intelligible fashion and written in standard English?

PLOS ONE does not copyedit accepted manuscripts, so the language in submitted articles must be clear, correct, and unambiguous. Any typographical or grammatical errors should be corrected at revision, so please note any specific errors here.

Reviewer #1: Yes

Reviewer #2: Yes

**********

5. Review Comments to the Author

Please use the space provided to explain your answers to the questions above. You may also include additional comments for the author, including concerns about dual publication, research ethics, or publication ethics. (Please upload your review as an attachment if it exceeds 20,000 characters)

Reviewer #1: The manuscript investigated the effects of etlyl ferulate (EF) in the modulation of inflammatory response in a model of murine macrophages and mice. The results have documented the capacity of EF to inhibit the inflammatory response both in vitro and in vivo.

Below are reported some comments to address

ABSTRACT:

-Please, replace TNFα with TNF-α

-I would suggest to change as follow: “inhibit the expression and production”

-Please, provide detailed results in terms of “effects” (how much increase and/or decrease?)

-Keywords should include also in vitro and in vivo.

INTRODUCTION:

- Please, provide a better explanation of the link between macrophages and acute lung injury. How macrophages, thus the in vitro model, is linked to the animal model. This would help the reader.

-In addition, provide some detail on the circulating levels commonly associated with ethyl ferulate when EF-rich foods are consumed and the concentrations of EF found in blood.

-Please, replace TNFα with TNF-α

- Please, define LPS, Nrf2

METHODS:

- Please, provide the catalogue number of material used in the experiments. For instance: ELISA kit, RNA extraction kit, Master Mix, Nuclear and Cytoplasmic Protein Extraction kit, Western blot antibodies.

- The authors should indicate the passage number that the cells were used to perform the experiments.

- Please, provide the different concentration of ethyl ferulate tested (expressed as molarity) and define if they are achievable in vivo.

- Please, explain the rationale to test 15 mg and 30 mg of ethyl ferulate.

- In order to promote experimental transparency and ensure consistency between laboratories, it is strongly suggested to present their qPCR data in line with MIQE Guidelines, including relevant QA Information on quality and amount of RNA extract.

RESULTS:

- I would suggest to improve results by reporting the magnitude of the effect and the statistics.

- Please, remove the definition of IL-6 and TNF-α since already reported in the introduction.

DISCUSSION:

- Authors should improve the discussion of the paper. In fact, despite the numerous findings obtained, the discussion is quite short/limited and it would benefit of an implementation.

- A comment on the ethyl ferulate doses is recommended. Are the doses tested comparable with others used by similar experiments? How to translate these doses to human?

- Conclusion should be revised in the form (e.g. please delete “fig 9”) and summarize better the main findings. In addition, a mention to the need of future studies devoted to understand/confirm the results obtained and/or elucidate some specific mechanisms should be postulated.

Reviewer #2: Dear Authors:

　I’m so grateful to review this manuscript namely” Ethyl Ferulate Contributes to the Inhibition of the Inflammatory Responses in Murine RAW 264.7 Macrophage Cells and Acute Lung Injury in Mice”, which introduces the anti-inflammatory effect of Ethyl Ferulate on LPS-induced Raw264.7 cell model and acute lung injury in mice. This research focusing the bioactive function of Ethyl Ferulate will produce a practical significance for the application of Ethyl Ferulate and its raw material. However, there are still some issues must be attention in your manuscript before acceptation, which are listed below:

The first part is about your experiments.

1、 what about the purpose of your animal experiment? you just talk about your in vitro experiment in your introduction, but nothing about your animal experiment. what's more, what about the relationship between your in vitro experiment and in vivo experiment?

2、 How long is your experiment period to finish your in vivo experiment? in other words, How long is the interval between your two injections of EF in mice?

3、 what is your absorb wavelength of your MTT assay? The wavelength must be shown.

4、 Why do you put the result of PGE2 and MTT together? The subtitle of your first part of your results is also little confused, for that most of the result below the first part is also about the effect of EF to the cells.

5、 Why dont you measure other pro-inflammatory cytokines like IL-1β, IL-2 in your in vitro experiment? In my opinions, the IL-1β has a vital role in inflammation formation. What’s more, the level of NO is also suggested to be measured, since you have measured the level of iNOS.

6、 In the assay using inhibitor ML385 to down regulate the Nrf2, why don't you use western blotting to verify the HO-1 protein expression just like what doing above? Moreover, How to explain this phenomenon in fig7, that EF can reduce ML385-induced TNF-α and IL-6 secretion? What is the possible mechanism?

7、 How to make sure that the LPS can affect the lung after nasal drip? Did other researchers use the same animal model in their researches? Please prove the rationally of this animal experiment.

8、 As to your in vivo experiment, why don’t you measure effect of EF on the NF-κB pathway and Nrf-2/HO-1 pathway in lung tissue? That is a great chance to verify the results you get in in vivo assay, so it is advised to add these experiments to make your manuscript more convincible.

The second part is about the figure in your manuscript. Most of the figure is not clear enough to read. Please replace the figures to high resolution versions. To the Part A of the figure 4, the imaging of the Histone H3 as internal reference is suboptimal, please replace a new one.

The final part is about your spelling ang grammar mistake in your manuscript: please unify the writing of some proper nouns in your manuscript such as NF-κB, Nrf2 and so on. Moreover, there are some spelling mistakes in your manuscript like mistaken “neutrophils” to “meutrophils”. Last but not the least, check the format of your manuscript to make sure there is no missing or additional characters in your manuscript.

**********

6. PLOS authors have the option to publish the peer review history of their article (what does this mean?). If published, this will include your full peer review and any attached files.

If you choose “no”, your identity will remain anonymous but your review may still be made public.

**Do you want your identity to be public for this peer review?** For information about this choice, including consent withdrawal, please see our Privacy Policy.

Reviewer #1: No

Reviewer #2: No

[NOTE: If reviewer comments were submitted as an attachment file, they will be attached to this email and accessible via the submission site. Please log into your account, locate the manuscript record, and check for the action link "View Attachments". If this link does not appear, there are no attachment files.]

While revising your submission, please upload your figure files to the Preflight Analysis and Conversion Engine (PACE) digital diagnostic tool, https://pacev2.apexcovantage.com/. PACE helps ensure that figures meet PLOS requirements. To use PACE, you must first register as a user. Registration is free. Then, login and navigate to the UPLOAD tab, where you will find detailed instructions on how to use the tool. If you encounter any issues or have any questions when using PACE, please email PLOS at figures@plos.org. Please note that Supporting Information files do not need this step.

# Author comment: Author response to Decision Letter 0

**DOI:** 10.1371/journal.pone.0251578.r002

26 Apr 2021

Dear editor,

We thank the reviewers and editor for their insightful comments. We have revised our

research paper titled, “Ethyl Ferulate Contributes to the Inhibition of the Inflammatory Responses in Murine RAW 264.7 Macrophage Cells and Acute Lung Injury in Mice” based on their useful suggestions. We believe that these modifications have further improved the manuscript. Our responses to the Reviewers’ suggestions/comments are as follows.

Reviewer #1: The manuscript investigated the effects of etlyl ferulate (EF) in the modulation of inflammatory response in a model of murine macrophages and mice. The results have documented the capacity of EF to inhibit the inflammatory response both in vitro and in vivo.

Below are reported some comments to address

ABSTRACT:

1. Please, replace TNFα with TNF-α

Thanks for the comment. We did it.

2. I would suggest to change as follow: “inhibit the expression and production”

Thanks for the comment. We have changed it.

3. Please, provide detailed results in terms of “effects” (how much increase and/or decrease?)

Thanks for the comment. EF could attenuate the degree of leukocyte infiltration, reduced MPO activity, mRNA levels and secretion of TNF-α and IL-6 in vivo.

4. Keywords should include also in vitro and in vivo.

Thanks for the comment. We did it.

INTRODUCTION:

5. Please, provide a better explanation of the link between macrophages and acute lung injury. How macrophages, thus the in vitro model, is linked to the animal model. This would help the reader.

Macrophages is the main pro-inflammatroy cells and regulation of macrophage polarization may be a potential rational therapeutic target for acute lung injury. Macrophages release various proinflammatory meditors, such as tumor necrosis factor α (TNF-α) and interleukin 6 (IL-6) following lipopolysaccharide (LPS) stimulation.

6. In addition, provide some detail on the circulating levels commonly associated with ethyl ferulate when EF-rich foods are consumed and the concentrations of EF found in blood.

EF presents in various systems of many plants, such as the solanaceas family as a trace contituent, which is more lipophilic and has higher cell membrane and blood-brain barrier permeability than FA.

7. Please, replace TNFα with TNF-α

Thanks for the comment. We did it.

8. Please, define LPS, Nrf2

Thanks for the comment. We did it.

METHODS:

9. Please, provide the catalogue number of material used in the experiments. For instance: ELISA kit, RNA extraction kit, Master Mix, Nuclear and Cytoplasmic Protein Extraction kit, Western blot antibodies.

Thanks for the comment. We did it.

10. The authors should indicate the passage number that the cells were used to perform the experiments.

All experiments were performed with cells on passages 6 to 10.

11. Please, provide the different concentration of ethyl ferulate tested (expressed as molarity) and define if they are achievable in vivo.

Different concentrations (10, 20, 40, 50, 80 mg/L) of EF were applied. The dose of ethyl ferulate is not used in animal experiment. The dose of animal experiment was obtained through literature research and pre experiment. 

12. Please, explain the rationale to test 15 mg and 30 mg of ethyl ferulate.

About the dose ethyl ferulate of animal experiment, we refer to the master's thesis for the dosage (Synthesis of Ethyl ferulate and study on its Pharmaeologieal aeitviites), and then we carried out the pre-experiment. Finally, we chose 15 mg and 30 mg of ethyl ferulate 

13.In order to promote experimental transparency and ensure consistency between laboratories, it is strongly suggested to present their qPCR data in line with MIQE Guidelines, including relevant QA Information on quality and amount of RNA extract.

We extracted the total RNA from the raw 264.7 macrophage cells, the A260/280 and A260/230 of RNA were analyzed by Micro ultraviolet visible absorption/ fluorescence photometer (Malcom ES-2). CON, A260/280:1.99, A260/230:2.13, LPS, A260/280:2.01, A260/230:2.18, LPS+EF, A260/280:1.89, A260/230:2.23, A260/280:2.02, A260/230:2.37.

RESULTS:

14. I would suggest to improve results by reporting the magnitude of the effect and the statistics.

Thanks for the comment. We did it.

15. Please, remove the definition of IL-6 and TNF-α since already reported in the introduction.

Thanks for the comment. We did it.

DISCUSSION:

16. Authors should improve the discussion of the paper. In fact, despite the numerous findings obtained, the discussion is quite short/limited and it would benefit of an implementation.

Thanks for the comment. We modified our discussion

17. A comment on the ethyl ferulate doses is recommended. Are the doses tested comparable with others used by similar experiments? How to translate these doses to human?

Through literature research, EF has aroused interest due to anti-inflammatory and antioxidant. Yung-Chieh et al, reported pretreatment of FAEE (1 and 10 μm) in A10 cells could significantly leptin-induced proliferation and migration of aortic smooth muscle cells (The effect of ferulic acid ethyl ester on leptin-induced proliferation and migration of aortic smooth muscle cells). To investigate whether EF could attenuate oxidative stress in the retina, Masayuki et al, found EF (0.02 or 0.01 mM) did not affect the viability of ARPE-19 cells. Exposure to 0.4 mM H2O2 for 1 h caused a 50–60% loss in cell viability, and treatment with EF attenuated this cell damage (Oral administration of ferulic acid or ethyl ferulate attenuates retinal damage in sodium iodate-induced retinal degeneration mice). According these researches, we chose different concentrations (10, 20, 40, 50, 80 mg/L) of EF and with or without LPS in murine RAW 264.7 macrophage cells. In this study, we found 1 μg/mL LPS have no effect on cell viability in raw 264.7 macrophage cells. while the concentrations 10, 20, 40, 50, 80 mg/L of EF no cellular toxicity in raw 264.7 macrophage cells stimulated by LPS. Thank you very much for your advice. In our study, we have only conducted experimental studies on cells and animals, and how to translate human body will be the focus of our next work. 

18. Conclusion should be revised in the form (e.g. please delete “fig 9”) and summarize better the main findings. In addition, a mention to the need of future studies devoted to understand/confirm the results obtained and/or elucidate some specific mechanisms should be postulated.

We adjusted fig 9 and modify the conclusion

Reviewer #2: Dear Authors:

　I’m so grateful to review this manuscript namely” Ethyl Ferulate Contributes to the Inhibition of the Inflammatory Responses in Murine RAW 264.7 Macrophage Cells and Acute Lung Injury in Mice”, which introduces the anti-inflammatory effect of Ethyl Ferulate on LPS-induced Raw264.7 cell model and acute lung injury in mice. This research focusing the bioactive function of Ethyl Ferulate will produce a practical significance for the application of Ethyl Ferulate and its raw material. However, there are still some issues must be attention in your manuscript before acceptation, which are listed below:

.0T0ISZhe first part is about your experiments.

1、 what about the purpose of your animal experiment? you just talk about your in vitro experiment in your introduction, but nothing about your animal experiment. what's more, what about the relationship between your in vitro experiment and in vivo experiment?

Dear reviewer, thank you very much for your advice. In our study, we preliminary analyzed the anti-inflammation effect of EF in acute lung injury mice. And the the possible mechanism of anti-inflammation effect of EF was examined in RAW 264.7 macrophages. Through investigation, Macrophages is the main pro-inflammatroy cells and regulation of macrophage polarization may be a potential rational therapeutic target for acute lung injury. Macrophages release various proinflammatory meditors, such as tumor necrosis factor α (TNF-α) and interleukin 6 (IL-6) following lipopolysaccharide (LPS) stimulation. So we used macrophages to set up acute lung injury related cell model. The inducer of animal and cells, we chose lipopolysaccharide (LPS). 

2、How long is your experiment period to finish your in vivo experiment? in other words, How long is the interval between your two injections of EF in mice?

Mice were intraperitoneally injected with two doses (15 and 30 mg/kg) of EF with vehicle twice a day for 5 day

3、what is your absorb wavelength of your MTT assay? The wavelength must be shown.

Thanks for the comment. wavelength: 570 nm

4、 Why do you put the result of PGE2 and MTT together? The subtitle of your first part of your results is also little confused, for that most of the result below the first part is also about the effect of EF to the cells.

Thanks for the comment. We have modified first part of our results. 

5、 Why dont you measure other pro-inflammatory cytokines like IL-1β, IL-2 in your in vitro experiment? In my opinions, the IL-1β has a vital role in inflammation formation. What’s more, the level of NO is also suggested to be measured, since you have measured the level of iNOS.

Thanks for the comment. Your advice help us, we analyzed the supernatant levels of IL-1β were identified by ELISA. Your advice helps us about the level of NO, we will measure the level of NO in further work. Thank you very much. 

6、 In the assay using inhibitor ML385 to down regulate the Nrf2, why don't you use western blotting to verify the HO-1 protein expression just like what doing above? Moreover, How to explain this phenomenon in fig7, that EF can reduce ML385-induced TNF-α and IL-6 secretion? What is the possible mechanism?

Thanks for the comment. We have analyzed the mRNA of ho-1, iNOS and COX-2 in RAW 264.7 macrophages cells treated with inhibition of Nrf2. In our results, the possible mechanism is EF inhibit NF-κB pathway and activate Nrf2/HO-1 pathway by regulation of inflammatory responses by Nrf2

7、 How to make sure that the LPS can affect the lung after nasal drip? Did other researchers use the same animal model in their researches? Please prove the rationally of this animal experiment.

Thanks for the comment. The Acute Lung Injury model, we chose LPS by nasal drip 1h later. We refer to the previous work of the laboratory (Master's thesis: Anti-inflammatory activities of compounds of N-2 and BDM and their underlying mechanisms of action)

8、 As to your in vivo experiment, why don’t you measure effect of EF on the NF-κB pathway and Nrf-2/HO-1 pathway in lung tissue? That is a great chance to verify the results you get in in vivo assay, so it is advised to add these experiments to make your manuscript more convincible.

Thank you very for your advice. In vivo experiment, we just proved the effectiveness of EF. The possible mechanism of anti-inflammation effect of EF was examined in RAW 264.7 macrophages. Your suggestion is of great help to us, and it is also the content of our future research.

9、 The second part is about the figure in your manuscript. Most of the figure is not clear enough to read. Please replace the figures to high resolution versions. To the Part A of the figure 4, the imaging of the Histone H3 as internal reference is suboptimal, please replace a new one.

Thanks for the comment. We have changed it.

10、The final part is about your spelling ang grammar mistake in your manuscript: please unify the writing of some proper nouns in your manuscript such as NF-κB, Nrf2 and so on. Moreover, there are some spelling mistakes in your manuscript like mistaken “neutrophils” to “meutrophils”. Last but not the least, check the format of your manuscript to make sure there is no missing or additional characters in your manuscript.

Thanks for the comment. We did it.

# Peer review: Decision Letter 1

**DOI:** 10.1371/journal.pone.0251578.r003

**Authors:** Yi Cao

29 Apr 2021

Ethyl Ferulate Contributes to the Inhibition of the Inflammatory Responses in Murine RAW 264.7 Macrophage Cells and Acute Lung Injury in Mice

PONE-D-21-04411R1

Dear Dr. gong,

We’re pleased to inform you that your manuscript has been judged scientifically suitable for publication and will be formally accepted for publication once it meets all outstanding technical requirements.

Within one week, you’ll receive an e-mail detailing the required amendments. When these have been addressed, you’ll receive a formal acceptance letter and your manuscript will be scheduled for publication.

An invoice for payment will follow shortly after the formal acceptance. To ensure an efficient process, please log into Editorial Manager at http://www.editorialmanager.com/pone/, click the 'Update My Information' link at the top of the page, and double check that your user information is up-to-date. If you have any billing related questions, please contact our Author Billing department directly at authorbilling@plos.org.

If your institution or institutions have a press office, please notify them about your upcoming paper to help maximize its impact. If they’ll be preparing press materials, please inform our press team as soon as possible -- no later than 48 hours after receiving the formal acceptance. Your manuscript will remain under strict press embargo until 2 pm Eastern Time on the date of publication. For more information, please contact onepress@plos.org.

Kind regards,

Yi Cao

Academic Editor

PLOS ONE

Additional Editor Comments (optional):

Reviewers' comments:

Reviewer's Responses to Questions

**Comments to the Author**

1. If the authors have adequately addressed your comments raised in a previous round of review and you feel that this manuscript is now acceptable for publication, you may indicate that here to bypass the “Comments to the Author” section, enter your conflict of interest statement in the “Confidential to Editor” section, and submit your "Accept" recommendation.

Reviewer #1: All comments have been addressed

**********

2. Is the manuscript technically sound, and do the data support the conclusions?

The manuscript must describe a technically sound piece of scientific research with data that supports the conclusions. Experiments must have been conducted rigorously, with appropriate controls, replication, and sample sizes. The conclusions must be drawn appropriately based on the data presented. 

Reviewer #1: Yes

**********

3. Has the statistical analysis been performed appropriately and rigorously? 

Reviewer #1: Yes

**********

4. Have the authors made all data underlying the findings in their manuscript fully available?

The PLOS Data policy requires authors to make all data underlying the findings described in their manuscript fully available without restriction, with rare exception (please refer to the Data Availability Statement in the manuscript PDF file). The data should be provided as part of the manuscript or its supporting information, or deposited to a public repository. For example, in addition to summary statistics, the data points behind means, medians and variance measures should be available. If there are restrictions on publicly sharing data—e.g. participant privacy or use of data from a third party—those must be specified.

Reviewer #1: Yes

**********

5. Is the manuscript presented in an intelligible fashion and written in standard English?

PLOS ONE does not copyedit accepted manuscripts, so the language in submitted articles must be clear, correct, and unambiguous. Any typographical or grammatical errors should be corrected at revision, so please note any specific errors here.

Reviewer #1: Yes

**********

6. Review Comments to the Author

Please use the space provided to explain your answers to the questions above. You may also include additional comments for the author, including concerns about dual publication, research ethics, or publication ethics. (Please upload your review as an attachment if it exceeds 20,000 characters)

Reviewer #1: The manuscript has been adequately revised according to my comments. For me, the paper can be accepted for pubblication in the present form

**********

7. PLOS authors have the option to publish the peer review history of their article (what does this mean?). If published, this will include your full peer review and any attached files.

If you choose “no”, your identity will remain anonymous but your review may still be made public.

**Do you want your identity to be public for this peer review?** For information about this choice, including consent withdrawal, please see our Privacy Policy.

Reviewer #1: No

# Editor report: Acceptance letter

**DOI:** 10.1371/journal.pone.0251578.r004

**Authors:** Yi Cao

7 May 2021

PONE-D-21-04411R1 

Ethyl Ferulate Contributes to the Inhibition of the Inflammatory Responses in Murine RAW 264.7 Macrophage Cells and Acute Lung Injury in Mice 

Dear Dr. Gong:

I'm pleased to inform you that your manuscript has been deemed suitable for publication in PLOS ONE. Congratulations! Your manuscript is now with our production department. 

If your institution or institutions have a press office, please let them know about your upcoming paper now to help maximize its impact. If they'll be preparing press materials, please inform our press team within the next 48 hours. Your manuscript will remain under strict press embargo until 2 pm Eastern Time on the date of publication. For more information please contact onepress@plos.org.

If we can help with anything else, please email us at plosone@plos.org. 

Thank you for submitting your work to PLOS ONE and supporting open access. 

Kind regards, 

PLOS ONE Editorial Office Staff

on behalf of

Dr. Yi Cao 

Academic Editor

PLOS ONE
